# Neuromodulation through brain stimulation-assisted cognitive training in patients with post-chemotherapy subjective cognitive impairment (Neuromod-PCSCI) after breast cancer: study protocol for a double-blinded randomised controlled trial

**DOI:** 10.1136/bmjopen-2024-096162

**Published:** 2025-05-21

**Authors:** Merle Rocke, Elena Knochenhauer, Friederike Thams, Daria Antonenko, Anna Elisabeth Fromm, Nora Jansen, Samaneh Aziziaram, Ulrike Grittner, Sein Schmidt, Antje Vogelgesang, Eva-Lotta Brakemeier, Agnes Flöel

**Affiliations:** 1 Department of Neurology, Universitätsmedizin Greifswald, Greifswald, Germany; 2 Berlin Institute of Health at Charite, Berlin, Germany; 3 Charite - Universitätsmedizin Berlin Institut fur Biometrie und Klinische Epidemiologie, Berlin, Germany; 4 Charite - Universitätsmedizin Berlin Klinik fur Neurologie mit Experimenteller Neurologie, Berlin, Germany; 5 Department of Psychology and Psychotherapy, Universität Greifswald, Greifswald, Germany; 6 German Centre for Neurodegenerative Diseases, Bonn, Germany

**Keywords:** CHEMOTHERAPY, Quality of Life, Cognition

## Abstract

**Introduction:**

Breast cancer is the most common form of cancer in women. A considerable number of women with breast cancer who have been treated with chemotherapy subsequently develop neurological symptoms such as concentration and memory difficulties (also known as ‘chemobrain’). Currently, there are no validated therapeutic approaches available to treat these symptoms. Cognitive training holds the potential to counteract cognitive impairment. Combining cognitive training with concurrent transcranial direct current stimulation (tDCS) could enhance and maintain the effects of this training, potentially providing a new approach to treat post-chemotherapy subjective cognitive impairment (PCSCI). With this study, we aim to investigate the effects of multi-session tDCS over the left dorsolateral prefrontal cortex in combination with cognitive training on cognition and quality of life in women with PCSCI.

**Methods and analysis:**

The Neuromod-PCSCI trial is a monocentric, randomised, double-blind, placebo-controlled study. Fifty-two women with PCSCI after breast cancer therapy will receive a 3-week tDCS-assisted cognitive training with anodal tDCS over the left dorsolateral prefrontal cortex (target intervention), compared with cognitive training plus sham tDCS (control intervention). Cognitive training will consist of a letter updating task. Primary outcome will be the performance in an untrained task (n-back task) after training. In addition, feasibility, safety and tolerability, as well as quality of life and performance in additional untrained tasks will be investigated. A follow-up visit will be performed 1 month after intervention to assess possible long-term effects. In an exploratory approach, structural and functional MRI will be acquired before the intervention and at post-intervention to identify possible neural predictors for successful intervention.

**Ethics and dissemination:**

Ethical approval was granted by the ethics committee of the University Medicine Greifswald (BB236/20). Results will be available through publications in peer-reviewed journals and presentations at national and international conferences.

**Trial registration number:**

ClinicalTrials.gov; NCT04817566, registered on 26 March 2021.

**STRENGTHS AND LIMITATIONS OF THIS STUDY**

- Randomized, double-blind, placebo-controlled design minimizes bias inintervention assessment.
- Structured cognitive training protocol ensures consistency in the intervention.
- Inclusion of a follow-up visit allows evaluation of long-term effects.
- Monocentric trial design may increase risk of bias.
- Limited sample size may reduce between-group comparability regarding covariates(education, baseline cognition, clinical characteristics).

## Introduction

With the development of new treatment options, the long-term survival rates of breast cancer patients are increasing.^1^ However, cancer patients may develop cognitive impairments following chemotherapy.^2 3^ Cognitive impairment in cancer patients can arise from the underlying cancer itself or as a consequence of various treatments. Evidence shows that more than 20% of patients have cognitive problems even before the start of chemotherapy, which suggests the possibility of the impact of systemic inflammatory responses caused by tumour growth. Also, psychological factors such as stress, anxiety and depression that occur after receiving a cancer diagnosis play a significant role in these early cognitive changes.^4 5^ Cognitive impairment has also been reported in a wide range of cancer types and can occur as a result of various treatment methods such as surgery, radiation therapy, hormone therapy, targeted therapies and immunotherapy.^6^ In addition, factors such as increasing age, genetic background, sociodemographic conditions and concomitant medication for other diseases can also increase the severity of this disorder and emphasise its complexity.^7^ Post-chemotherapy cognitive impairment (PCCI), also referred to as ‘chemobrain’, can persist for many years after chemotherapy and occurs in various cognitive domains, such as executive functions, memory, processing speed and attention.^3 8^ Beyond that, cognitive impairment may cause psychological distress and affect quality of life.^9 10^

The pathophysiology of PCCI remains poorly understood. Multiple mechanisms underlying patterns of cognitive impairment in cancer patients have been proposed, including neuroinflammation, direct neurotoxicity, as well as alterations in structure, function and metabolic profile of the brain,^11^,^13^ as well as changes in neurotransmitters, such as reduced concentrations of norepinephrine, dopamine and serotonin, among others.^2 14 15^

Thus far, no evidence-based pharmacological treatments are available for PCCI.^16^ Although agents like donepezil^17^ and methylphenidate^18^ have been studied in randomised controlled trials, these studies have not shown significant improvements in cognitive function compared with a placebo, and neither of these agents has been approved. Due to potential side effects and the lack of robust evidence for pharmacological means, non-pharmacological interventions have gained increasing attention and popularity. Many studies have shown that non-pharmacological interventions such as cognitive rehabilitation, physical activities, mindfulness therapies, cognitive-behavioural therapy (CBT) and acupuncture can be effective in improving cancer-related cognitive impairment in breast cancer survivors.^16 19 20^ Meta-analyses and systematic reviews have reported beneficial effects of physical exercise,^21^ mindfulness-based interventions,^22^ and particularly cognitive training^23^ on both subjective cognitive complaints and objective cognitive performance. CBT has demonstrated efficacy in alleviating psychological distress and improving self-perceived cognitive functioning,^24^ while interventions such as yoga and aerobic exercise have shown additional emotional and cognitive benefits.^25 26^ Acupuncture has also been investigated as a complementary method, with promising results.^27^

Vance *et al*^28^ demonstrated that cognitive training interventions and compensatory strategies led to improvements in cognitive deficits among breast cancer survivors who had received chemotherapy. Similarly, a systematic review by Oldacres *et al*^29^ emphasised that cognitive training is among the most widely studied and promising interventions for cancer-related cognitive impairment.

First studies have found preliminary evidence that cognitive training may improve cognitive abilities of patients with PCCI.^30^,^32^ Von Ah *et al* observed an improvement in trained functions and a transfer effect to untrained memory tasks as well as to self-rated subjective cognitive function and reduced psychological stress in the trained group compared with a waiting list control group after training of memory and speed of processing.^31^ Similarly, in the study of Kesler *et al* participants trained cognitive flexibility, working memory, processing speed and verbal fluency and subsequently showed enhanced performance in cognitive functions mediated by the prefrontal cortex as well as minor improvements in declarative memory scores.^32^ In a meta-analysis by Yan *et al*,^23^ nine randomised controlled trials (RCTs) involving a total of 666 patients with breast cancer were reviewed to assess the effectiveness of cognitive training in patients with cognitive changes. The evidence suggests that cognitive training may improve subjective cognitive function, processing speed, verbal memory, working memory and episodic memory in patients with breast cancer who are reporting cognitive changes.

Despite these promising preliminary findings, training programmes have been time-intensive, involving several weeks of task practice, and transfer effects have not consistently been demonstrated.^33^

Non-invasive brain stimulation is an approach to boost training gains, which allows for shorter training periods and for induction of transfer to untrained functions. Transcranial direct current stimulation (tDCS) is an inexpensive, painless method that is well-tolerated, safe and easy to apply.^34^ This promising approach is already widely used for interventional and therapeutic purposes (see Filmer *et al*^35^ for review). For the administration of tDCS, a weak electric current is applied via two or more electrodes on the participant’s scalp. The supposed mechanism of action is a directional modulation of the cortical excitability in the brain area under the electrodes as well as in functionally and structurally connected brain regions.^36^ The application of direct current causes a shift in the resting membrane potential of the cortical neurons, which in turn leads to a decrease in the threshold for triggering action potentials. Accordingly, the corresponding area of the brain shows a higher level of excitability. Furthermore, the excitatory effect of anodal stimulation leads to an increase in synaptic transmission, which can last for hours to days. This effect corresponds to a long-term potentiation-like mechanism and can induce neural plasticity.^37^ Promising results of the technique with regard to improving cognitive performance have been demonstrated in healthy ageing, but also in patients with dementia or mild cognitive impairment (MCI).^38^,^42^

To our knowledge, no study to date has examined the effects of tDCS-assisted cognitive training in patients with PCCI in order to establish its potential to beneficially impact cognitive functions and patient-reported outcome measures such as health-related quality of life (HRQoL). In the current study, we plan to assess in a double-blind randomised controlled phase II clinical trial if such a combined multi-session cognitive training plus tDCS intervention yields substantial long-term benefits and transfer effects in patients with subjective cognitive impairment after chemotherapy, so-called post-chemotherapy subjective cognitive impairment (PCSCI). All patients will complete cognitive training of a letter updating task over nine training sessions with concurrent tDCS over the left dorsolateral prefrontal cortex (dlPFC). Half of the sample will receive anodal tDCS while performing the cognitive training, whereas the other half will undergo sham stimulation during training. The intervention will span 3 weeks, with three training sessions per week. We will assess behavioural outcome measures, such as direct training effects, feasibility, safety and tolerability, transfer to untrained domains and long-term effects on cognition and quality of life at multiple time points. We will further elucidate neural characteristics associated with interventional success before the intervention and assess neural correlates at post-intervention. This protocol, describing the design and methods of the Neuromod-PCSCI study, was prepared in accordance with the SPIRIT guidelines.^43 44^ With regard to both feasibility and behavioural outcomes, the purpose of the present study is to inform planning and guide protocol development (including power analysis) in relation to future, confirmatory randomised controlled trials (RCTs) on the efficacy of cognitive training-plus-tDCS in increasing cognitive functions in patients with PCSCI.

## Methods: participants, intervention and outcomes

### Design and setting

**Figure 1:**
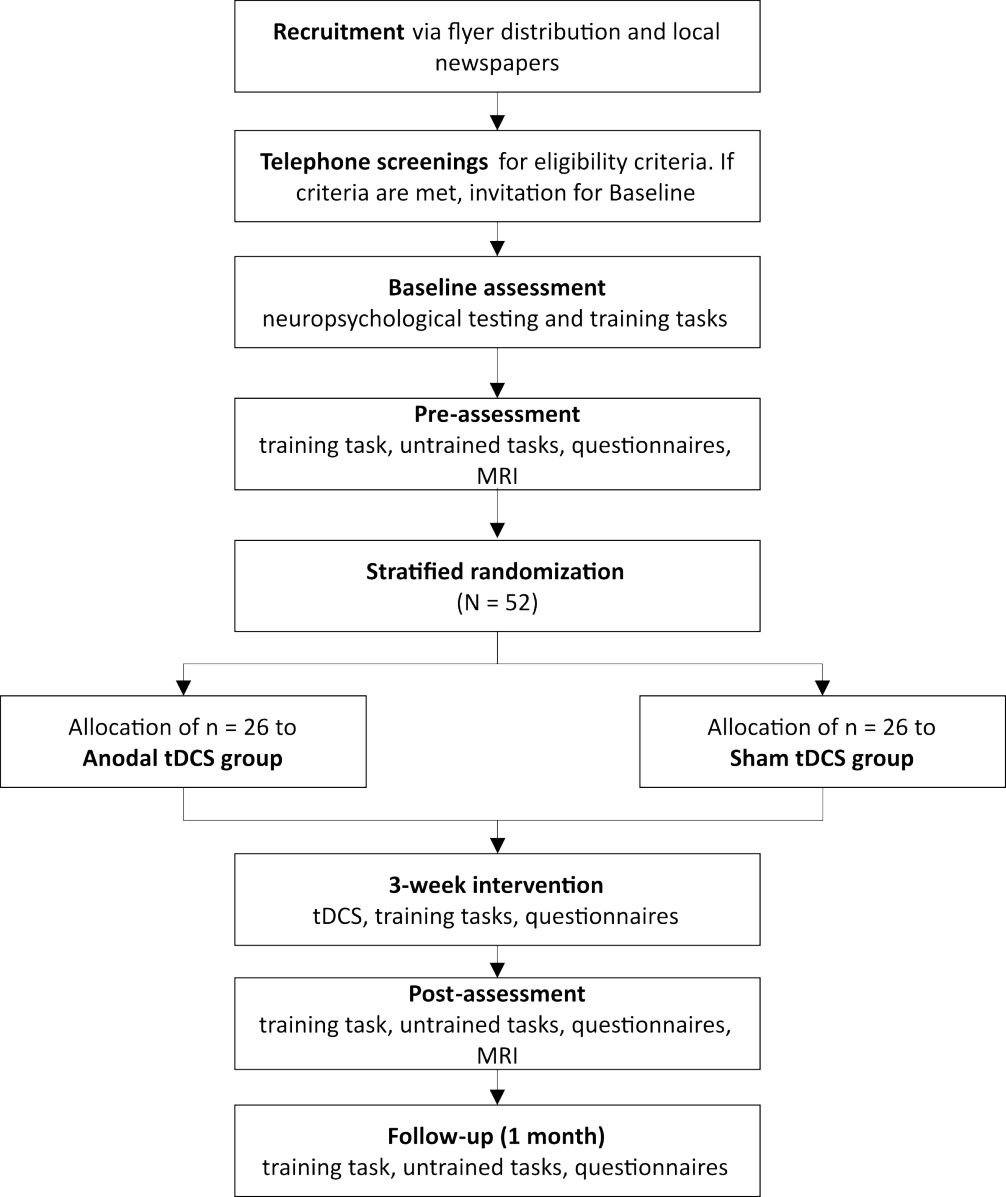
Neuromod-PCSCI study flowchart. tDCS, transcranial direct current stimulation; ZPP, Zentrum für Psychologische Psychotherapie (university psychotherapy outpatient clinic).

This is a monocentric, randomised, double-blind, placebo-controlled study. The intervention is composed of nine sessions of cognitive training over 3 weeks, accompanied by anodal tDCS over the left dlPFC compared with sham tDCS. Subjects will participate in 13 sessions altogether with pre-intervention and post-intervention assessments. One follow-up session is planned 1 month after the intervention to assess possible long-term effects. All sessions will take place at the University Medicine Greifswald, Greifswald, Germany. MRI will be performed before the intervention and at post-intervention (‘add-on’ study, that is, optional for the participants). The first participant was enrolled on 2 November 2021 and the anticipated end of the study will be the end of June 2026. This trial was prospectively registered at ClinicalTrials.gov (identifier: NCT04817566, registered 26 March 2021). A flowchart of the study is shown in figure 1.

### Eligibility criteria

Participants included in the study must meet all of the following inclusion criteria:

- Chemotherapy to treat breast cancer (over 6 months since end of treatment)
- Self-reported concerns regarding cognitive function
- Age: 18–65 years
- Right-handedness

In case one or more of the following criteria are present at randomisation, potential participants will be excluded:

- History of dementia before treatment of cancer
- Other neurodegenerative neurological disorders, epilepsy or a history of seizures
- Severe and untreated medical conditions that preclude participation in the training, as determined by responsible physician
- History of moderate to severe substance use disorder according to DSM-5^45^
- Moderate to severe acute mental disorders according to DSM-5^45^
- Contraindication to tDCS^34^

Participants include right-handed women (18–65 years) with chemotherapy-treated breast cancer (completed ≥6 months prior), excluding those with other cancers, current malignancies or pre-treatment cognitive complaints. This window captures both subacute and chronic post-chemotherapy effects while controlling for major confounders through strict neurological/psychiatric exclusions. We restricted participation to right-handed individuals due to well-documented hemispheric differences in cognitive processing and tDCS response, and our stimulation target exhibits more consistent functional organisation in right-handed individuals,^46 47^ reducing variability in neuromodulation effects. This approach also minimises confounds from interhemispheric reorganisation patterns more common in non-right-handed populations. Note that contraindication to MRI will not be treated as exclusion criteria as participants will still be included in the study sample, but no MRI scans will be performed in these individuals. If all eligibility criteria are met and participants provide written informed consent (online supplemental material), they will be included in the study sample.

### Intervention

At each of the training sessions, participants will receive either anodal or sham tDCS while completing a cognitive training task. Before starting the training task, the scalp of the participants will be numbed via an application of a topical anaesthetic (EMLA

Cream, 25 mg/g Lidocain+25 mg/g Prilocain). The EMLA cream will be applied on the scalp according to the positioning of the electrodes and will be left to take effect for about 20 min. After the exposure time, the EMLA cream will be removed, the tDCS setup will be mounted and stimulation will be started.

Participants will be presented with a letter updating (LU) task on a tablet computer to train working memory updating. List of letters A to D (with lengths of 5, 7, 9, 11, 13 or 15 letters, six times each; total of 36 lists) will be presented in random order, one letter at a time (presentation duration 2000 ms, ISI 500 ms). After each list, participants will be asked to recall the last four letters that were presented.

TDCS will be administered via a battery-operated stimulator (Neuroelectrics Starstim 8, Barcelona, Spain). A current of 2 mA will be applied via five round electrode-gel filled NG PigStim electrodes (NE029, Neuroelectrics, Barcelona, Spain), connected to the stimulator and mounted in a neoprene head cap using the 10–20 EEG grid. The stimulation will be applied continuously for 20 min, with 20 additional sec of ramping at the beginning and at the end of stimulation, respectively. The anodal electrode will be placed over the left dlPFC (F3). The four cathodal electrodes will be arranged in a circle around the anodal electrode, in the positions of AFz, F7, C5 and FCz, respectively, to constrain the current flow to the target region. In the sham group, current will be applied for 30 s to blind the participants regarding their stimulation condition. Ramp times and electrode montage will be the same as in the anodal group. The cognitive training task and the stimulation will be started simultaneously. After every third session, participants will fill out an adverse-events questionnaire.^34^ Participants will be instructed to avoid excessive consumption of alcohol (< 0.1 L of wine) the day before the intervention and to abstain from all alcohol consumption on the day of the intervention. Nicotine users are permitted to maintain their typical consumption patterns to prevent withdrawal-related artefacts but are monitored for deviations. These criteria are reinforced through pre-intervention questionnaires, and individuals with a history of alcohol/drug abuse are excluded during screening. Moreover, participants will be asked to adhere to their regular sleep schedule and to avoid drinking caffeine 90 min prior to the training sessions.

### Outcome measures

#### Primary outcomes

Primary outcome measure will be working memory performance at post-assessment, operationalised by the percentage of correct responses in the n-back task (untrained task).

#### Secondary outcomes

Secondary outcome will be working memory performance in the untrained task (n-back,^48^ at follow-up assessment, operationalised by the percentage of correct responses). The n-back task (1-back and 2-back) will assess working memory by requiring participants to indicate via keypress whether the current digit matches the one presented ‘n’ steps earlier. This untrained task evaluates updating, discrimination and decision-making processes (detailed methods in Neuropsychological assessment). Additionally, performance in the primary memory training task (LU task)^49^ and visuospatial memory performance (virtual reality (VR) task)^50^ will be assessed at post and follow-up assessments. Performance in the training task will be operationalised by number of correctly recalled lists in the LU task, and visuospatial performance will be operationalised by number of correctly recalled items in the VR task.

Further secondary outcome measures include quality of life, operationalised via the PROMIS Preference score (PROPr score) which serves as a summary scoring system of health-related quality of life including domains such as depression, fatigue, pain interference and ability to participate in social roles and activities.^51^ The domain of cognitive function will be scored separately because it is the most important subscale for this study. Scoring will be carried out according to the scoring manuals (http://www.healthmeasures.net/). Feasibility will be operationalised through drop-out rates (% participants that did not complete post-assessment), adherence rates of training sessions (number of training sessions attended) and the post-study system usability questionnaire (PSSUQ),^52^ completed by study assessors. Additionally, structural and functional neural correlates (assessed at pre-assessments and post-assessments), as measured by structural and functional MRI, will be assessed.

### Exploratory analyses

Exploratory analyses will be conducted for more detailed outcomes of the training task (eg, outcomes dependent on list length in the LU task). Additionally, education, baseline cognitive ability or neuropsychological status, as well as sociodemographic and clinical characteristics, will be analysed to identify potential predictors of training task performance and response to the intervention. Moreover, learning-relevant polymorphisms as well as (neuro-)inflammatory markers in collected blood samples will be analysed to examine mechanisms underlying PCCI pathology and their link to beneficial training gains induced by the intervention.

### Participant timeline

**Figure 2:**
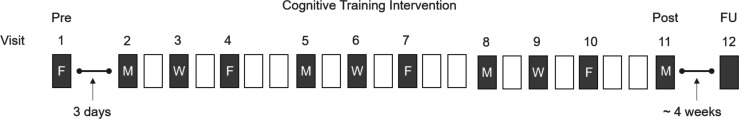
Participant timeline. Individuals will participate in 13 visits and two optional MRI sessions, taking place at the University Medicine Greifswald. After inclusion at the baseline visit (**V0**), participants will attend the pre-assessment visit (on a Friday, **V1**) before commencing the nine training visits, which will take place over 3 consecutive weeks, 3 days a week (**V2–V10**). The cognitive training intervention will take place on Monday (**M**), Wednesday (**W**) and Friday (**F**). After the training, a post-assessment (on a Monday, **V11**) will be conducted, and 4 weeks later, a follow-up visit (**V12**) will be administered. MRI will be acquired before pre-assessment (**V1**) and at post-assessment (**V13**). F, Friday; M, Monday; T, Tuesday.

Individuals will participate in 13 visits and two optional MRI sessions, taking place at the University Medicine Greifswald. After inclusion at baseline visit (V0), participants will attend the pre-assessment visit (V1, Friday) before starting the nine training visits during three consecutive weeks on 3 days a week (V2–V10). The cognitive training intervention will take place on Monday (M), Wednesday (W) and Friday (F). After the training, a post-assessment (V11, Monday) will be conducted and 4 weeks later a follow-up visit (V12) will be administered. MRI will be acquired before pre-assessment (V1) and at post-assessment (V13). Figure 2 provides a concise overview of this participant timeline.

### Baseline measures

At baseline, participants will provide written informed consent (online supplemental material) and participate in a demographic interview. The participants will be asked to provide a record of their cancer-specific medical history. If study-relevant information is missing, it will be requested from the responsible physician. Furthermore, a diagnostic interview to screen for mental disorders will be conducted (see section: Psychological assessment). Subsequently, the participants will be tested with a comprehensive battery of neuropsychological tests to quantify cognitive function on different domains (see section: Neuropsychological assessment). For more details, please refer to the following corresponding subsection.

Afterwards, participants will perform the training task as described above, with the exception that at baseline the task will consist of one practice trial with four lists and one actual training trial with 25 lists (compared with 36 lists in the training). The baseline assessment will have an approximated duration of 3,5 hour.

An actigraph (GT3X, ActiGraph, Pensacola, FL, USA) will be handed out to the participants at baseline to record sleep and activity data. Participants will be instructed to wear the device in the week leading up to pre-assessment and in the week following the post-assessment. Additionally, at baseline, participants will be asked to complete two sleep questionnaires: the Epworth Sleepiness Scale to measure daytime sleepiness^53^ and the Morningness-Eveningness Questionnaire^54^ to estimate the participant’s circadian rhythm.

### Pre, post and follow-up assessments

All three sessions will follow the same procedure. First, self-reported well-being, quality and duration of sleep, as well as potential stressors up to 2 hours prior to the visit, will be assessed by the investigator via a questionnaire. The participants will further complete an array of QoL-related questionnaires along with sleep questionnaires (see section: Psychological assessment). Then, participants will complete the training task and untrained tasks. The follow-up assessments will provide the possibility for assessing the maintenance of training and transfer effects.

### Sample size

Power calculation is based on recent studies using multisession application of cognitive training compared with a control training on immediate performance in the trained task (primary outcome).^55^,^57^ Based on these data, we estimated an effect size of 0.7 (Cohen’s d). To demonstrate an effect in the primary outcome between cognitive training groups and control (% correct in the n-back task for the anodal vs sham stimulation group) with an independent t-test using a two-sided significance level of α=0.1 and a power of at least 80 %, 52 participants (26 for target intervention group, 26 for control intervention group) need to be included. This conservative approach using a t-test was chosen for sample size estimation, even though we intend to analyse the primary outcome conducting an analysis of covariance (ANCOVA) model.^58^ This monocentric clinical trial will serve to calculate sample size for a subsequent multicentre clinical trial. Sample size estimation was conducted using R software (http://www.R-project.org) and the pwr package (https://cran.r-project.org/package=pwr). To account for approximately a 13% drop-out during the intervention phase, the total number to recruit is 60 (30/group).

### Recruitment

The participants will be recruited via distribution of flyers at the university psychotherapy outpatient clinic (Zentrum für Psychologische Psychotherapie) and at local psychotherapeutic and gynaecologist doctor’s offices in Greifswald and surroundings. Furthermore, we will search for participants via articles and announcements in the local newspaper. Telephone screenings assessing inclusion and exclusion criteria will be conducted with all potential participants, and study information will be provided. Eligible participants will be invited for baseline assessment.

### Methods: randomisation and blinding

#### Randomisation

Allocation of the participants to the experimental groups will be performed by a researcher not involved in the study. Participants will be randomly allocated in a 1:1 ratio to the two groups (anodal vs sham tDCS). Baseline performance in the n-back task will be used as strata (two performance strata; ≤87% correct and >87% correct in the n-back task). This 87% cut-off was empirically derived from prior studies using identical n-back paradigms,^59^ ensuring balanced distribution of high-performing and low-performing individuals across groups while maintaining sensitivity to detect stimulation and training effects at the group level. Randomisation blocks with varying block sizes will be generated for each of the two stimulation groups, using R software (https://www.R-project.org) and the blockrand package (http://CRAN.R-project.org/package=blockrand). Participants will then be allocated to the anodal or sham tDCS group based on the generated randomisation sequences within each block and stratum.

#### Blinding

As this is a double-blind trial, participants and investigators will be blinded regarding the stimulation condition. In the sham group, the current will be applied for 30 s to blind the participants to the intervention. In previous studies, sham tDCS has been shown to be a safe and valid method of blinding participants.^60^,^63^ At the end of the nine-session intervention, participants will be asked to state if they believe they received anodal or sham stimulation. As for the investigator blinding, a staff member who is not involved in this project will perform the randomisation according to the procedure described above and provide unidentifiable labels for the two stimulation protocols (anodal, sham) for the assessor. The assessor blinding will be ensured by using NIC software (https://www.neuroelectrics.com/), where the assessor will decide between ‘Protocol A’ and ‘Protocol B’ that corresponds to the participants ID number and will be able to apply the stimulation accordingly without knowledge of the respective stimulation condition.

### Methods: data collection, management and analysis

#### Data collection methods

Neuropsychological, behavioural, sleep data, as well as MRI data and blood samples will be collected from each participant. Study investigators will be thoroughly trained in administering the assessments. Additionally, participants will receive in-depth training on the handling of the Actigraph so that they can adequately and safely administer the sleep recording on their own. The questionnaires that are handed out to the participants will be explained by an investigator and will be accompanied by additional written instructions on how and when to complete the questionnaire. Time points and methods of data collection are shown in table 1. MRI data will be collected for individuals participating in the ‘add-on’ MRI study (optional) unless there are contraindications to MRI scanning.

**Table 1:** Neuromod-PCSCI outcome measures

Time point	Measurement	Mode	Baseline	Pre	T1-T9 (3 weeks)	Post (3 days)	FU (1 month)
			∼3 hours	∼2 hours	∼1 hours	∼2 hours	∼2 hours
			V0	V1	V2–V10	V11	V12
Enrolment							
Eligibility screening		Paper	x				
Informed consent		Paper	x				
Neuropsychological screening	Demographic data	Paper	x				
	CTS^69^	Paper	x				
	AVLT^64^	Paper	x				
	Digit Span^48^	Paper	x				
	Stroop^65^	Paper	x				
	TMT A/B^66^	Paper	x				
	ROCF^67^	Paper	x				
Quality of life	PROMIS^51^	Paper		x		x	x
	EORTC QLQ C30^71^	Paper		x		x	x
	EORTC BR23^71^	Paper		x		x	x
	FACT Cog^72^	Paper		x		x	x
Sleep data	Epworth Sleepiness Scale^53^	Paper	x				
	Pittsburgh Sleep Quality Index^73^	Paper		x		x	
	Morningness-Eveningness-Questionnaire^54^	Paper	x				
	Actigraph	Device		x		x	
Intervention					⟷		
Training task	Letter updating^49 92^ 93	Tablet-Computer	x	x	x	x	x
Brain stimulation	tDCS (anodal vs sham)	Device			x		
Questionnaires	Self-reported well-being	Paper		x	x	x	x
	PANAS^93^	Paper			x		
	Adverse Events Questionnaire^34^*	Paper			x		
Additional assessments							
Untrained tasks	n-back (primary outcome)^48^	Computer		x		x	x
	Virtual reality task^50^	Computer		x		x	x
Physical measures	MRI, optional			x		x	
	Blood draw		Once at any of these sessions				
Feasibility	Adherence rates	Paper			x	x	

All measures were acquired on site, except for screening which was done via telephone.

* Assessed only at the end of each training week (V4, V7 and 10).

AVLT, German version of the auditory verbal learning test; CTS, childhood trauma screener; EORTC QLQ C3/BR23, European Organization for Research and Treatment of Cancer, Quality of Life Questionnaire (QLQ C30), supplemented with the extension regarding breast cancer specific quality of life (BR23); FU, follow-up assessment; PANAS, positive and negative affect schedule; PROMIS, patient reported outcomes measurement system; ROCF, Rey-Osterrieth complex figure test; tDCS, transcranial direct current stimulation; TMT A/B, trail making test A/B; T1-T9, training 1-9; V0-V12, visits 0-12.

#### Neuropsychological assessment

Neuropsychological tests at baseline visit (V0) comprise paper-pencil as well as computer-based assessments. Performance in several cognitive domains will be tested with the auditory verbal learning test,^48 64^ the Digit Span,^48^ the Stroop test,^48 65^ the Trail Making Test A/B,^66^ and the Rey-Osterrieth Complex Figure Test.^67^

Trained and untrained tasks include paper-pencil and computer-based assessments. Detailed description of the training task is provided in the interventions section. At pre, post and follow-up sessions (V1, V11 and V12), the untrained tasks will be administered: Participants will perform a numeric n-back task (1 and 2 back) and a VR navigation task.^50^ The n-back (1 and 2 back) task will be administered as an untrained task to assess working memory function (18 trials total, 9 trials 1-back and 9 trials 2-back with 10 items each, presentation duration 1500 ms, ISI 2500 ms). The participants will have to state for a sequence of numerical digits presented one after the other, if the stimulus that is presented currently is the same stimulus as ‘n’-steps back. The participants will answer via a keypress, left arrow key for ‘yes’ if the stimuli match and right arrow key for ‘no’ if the stimuli differ. This task requires that different brain functions are coordinated effectively including updating, discrimination and matching of the stimuli and decision making.^48 68^ The VR task^50^ will be administered as an untrained task. Here, during encoding, participants will be instructed to memorise a route with several targets (eg, butcher, doctor’s office and grocery store); during subsequent recall, the participants will be asked to navigate the shortest route to given targets.

#### Psychological assessment

A variety of questionnaires will be administered over the course of the study. At the baseline visit (V0), the participants will be screened for childhood maltreatment and various mental disorders via the CTS^69^ and the DIPS.^70^ The CTS is a 5-item questionnaire regarding experiences of neglect and abuse in childhood and adolescence. The DIPS is an established diagnostic interview which helps to thoroughly explore different possible mental diagnoses. It guides the interviewer, covering the different aspects of each disorder, the degree of symptom expression, and subsequently aids in classifying the possible mental disorders according to DSM-5.

At pre, post and follow-up assessments, the following Quality of Life Questionnaires will be administered:

- Patient Reported Outcomes Measure System (PROMIS, https://promis-germany.de/) to assess physical, mental and social health
- EORTC QLQ C30,^71^ to measure QoL of cancer patients
- EORTC BR23, which is a module of the EORTC QLQ C30 and focuses on the mental and physical health of breast cancer patients
- FACT Cog,^72^ to gain additional insights on perceived cognitive impairments in cancer patients with chemotherapy-induced cognitive problems

Simultaneously with the collection of sleep data through actigraphy, the participants will be asked to fill in three questionnaires regarding their sleep quality and rhythm. The Morningness-Eveningness Questionnaire^54^ and the Epworth Sleepiness Scale^53^ will be administered at Baseline assessment. The Pittsburgh Sleep Index^73^ will be administered both at pre-assessment and post-assessment, assessing day-time sleepiness, quality of sleep and the sleep rhythm of the participants in the last month/weeks.

#### Magnetic resonance imaging

MRI will be acquired at the Baltic Imaging Center (Center for Diagnostic Radiology and Neuroradiology, Universitätsmedizin Greifswald) with a 3-Tesla scanner (Siemens Verio) using a 32-channel head coil, prior to the training intervention and at post-intervention. A T1-weighted 3D sequence, a 3D FLAIR, diffusion tensor imaging (DTI) and a resting-state fMRI sequence will be recorded. Additional T1-weighted and T2-weighted structural images will be acquired with parameters optimised for computational modelling to calculate electric field distributions (simnibs.org).^74 75^ Seed-based resting-state functional connectivity within and between large-scale task-relevant networks (eg, frontoparietal and default mode network) will be performed^55 76 77^ using the CONN toolbox (www.nitrc.org/projects/conn).^78^ White-matter pathways will be reconstructed from diffusion-weighted images using the TRACULA pipeline^79^ in Freesurfer (http://surfer.nmr.mgh.harvard.edu/), in order to extract tract fractional anisotropy and mean diffusivity.^80^,^83^ Changes in FA and MD on whole-brain level will be analysed using FSL’s tract-based spatial statistics (TBSS, www.fmrib.ox.ac.uk/fsl).^84 85^ MD in grey matter will also be explored for examination of intervention-induced microstructural grey matter change.^86^ Segmentation on high-resolution T1 scans will be performed to assess the volume of cortical and subcortical grey matter^49 87^ using the computational anatomy toolbox (CAT12, http://www.neuro.unijena.de/cat/) and Freesurfer (http://surfer.nmr.mgh.harvard.edu/).

#### Blood draw

At one of the sessions, a peripheral blood sample for conducting genetic analyses will be collected, preprocessed and stored at the Neuroimmunology Lab of University Medicine Greifswald, using cryo-preservation method. 2.5 mL of EDTA blood will be collected for analysing genetic polymorphisms relevant for learning processes such as ApoE4 or BDNF. In addition, 5 mL of peripheral blood will be withdrawn to gain 2.5 mL of serum for biomarker analyses to gain information on (neuro-)inflammatory processes. We will quantify NAA/Cho, anti-NMDA-antibodies and VILIP-1, CCL2 (MCP-1), sTREM-2, BDNF, TGF-β1, VEGF, IL-6, sTREM-1, β-NGF, IL-18, TNF-α, sRAGE, CX3CL1 (Fractalkine), IL-1b and IL-10. Biomarker analyses will be performed at the Department of Neurology, Greifswald. Here, pseudonymised blood samples will be stored at −80°C until analysis. Rest material will be stored for up to 10 years. Analyses of anti-neural-antibodies will be performed at Labour Krone GbR in Bad Salzulfen, Germany. Genetic analyses will be performed at the Institute of Pharmacology at the University Medicine Greifswald.

#### Retention and adherence

To ensure adherence and retention throughout the entire study period, participants will be contacted regularly via telephone or email and will be provided with information about their appointments. A few days prior to the intervention, a member of the study team will call the participant to remind them of the upcoming appointments and to check if there are any open questions to address. Additionally, the participants will be provided with a document listing all their appointments with notes regarding parking and caffeine consumption at pre-assessment. At the end of each visit, the participants will be reminded of the date and time of the next session. Throughout the whole study period, participants will be encouraged to contact the 24/7 study answer machine if questions or concerns arise. Similarly, they will be prompted to leave a voice message if they are unable to attend or wish to reschedule a session. They then will be contacted to discuss alternative scheduling or to address possible questions and concerns. At the end of the study, participants will receive a reasonable financial reimbursement (approximately 10 € per hour) and the results of their neuropsychological screening and, if they underwent MRI scanning, their structural MRI on a compact disc. If complete adherence to the protocol is not possible, any effort to collect as much data as possible will be made.

### Data management and monitoring

All participant data will be pseudonymised. Spreadsheets containing both participant IDs and personal data will be protected with a password only known to the study staff and will be stored on a secure file server. Similarly, digital data, that is, output files from computer-based tasks, will be saved on a secure file server directly after acquisition. Non-digitally acquired data will be manually digitalised by a staff member of the research team and double-checked by another member. Progress of data entry and checking procedures will be documented. As additional quality control, we implemented systematic quality control by checking for outliers or missing values in key outcomes on a weekly basis and cross-checking 100% of critical documents (consent forms, adverse events). Additionally, protocol adherence is further monitored through equipment logs and weekly team meetings. Files containing subject records will be sorted by participant ID and stored securely. Sensitive data, such as names and medical records, will be scored separately in lockable cabinets in rooms with access restricted to the researchers. Protocols of the tDCS setup of each participant and session will also be stored on the file server, and MRI data will be pseudonymised before analysis. Following good scientific practice, data will be stored for at least 10 years.

### Adverse events monitoring

Safety and tolerability will be assessed through monitoring any potentially occurring adverse events (AEs) via an adverse events questionnaire,^34^ administered at the end of each training week (V4, V7 and V10). We will refrain from administering the AE questionnaire at every visit, since this would possibly draw the participant’s attention to minor sensations during the stimulation and would only serve as a distraction from the training task. Generally, adverse events during tDCS are rare and minimal. Known AEs with the applied stimulation parameters (20 min, 2 mA) are skin reddening, a tingling sensation under the electrode sites and occasionally a mild headache.^34^ As we will be using a local anaesthetic under the electrode sites, we expect even less noticeable sensations.^88 89^ Investigators will be instructed to monitor AEs and serious AEs (SAEs) throughout the study and document all detected AEs and SAEs. Participants will be informed at baseline assessment about all possible AEs and risks and can withdraw their consent at any time without providing a reason. In case an SAE occurs, the study physician will first make an assessment as to whether a causal relationship with the intervention is considered plausible. If more than three of the enrolled participants suffer from SAEs likely to be associated with the intervention (as assessed by the study physician), the trial will be discontinued.

## Statistical methods

The primary outcome, percent of correct responses in the n-back task at post-training compared with the pre-training assessment, will be analysed using ANCOVA with post-assessment n-back performance as dependent variable, pre-assessment n-back performance as covariate and group allocation (target intervention (n=26) vs control intervention (n=26)) as independent variable. Linear mixed models will be conducted for secondary outcomes with experimental group as a between-subjects factor. All models will be corrected for age and performance at pre-assessment. We will use random intercept models that account for the clustering of measures within individuals. In case of violation of requirements for parametric methods, data will be transformed before analysis. Analyses of primary and secondary outcomes will be reported in detail in the statistical analysis plan to be written and registered before unblinding of investigators performing the analyses. Confirmatory analysis of treatment effects will be conducted within an intention to treat (ITT) framework with multiple imputed data sets in case of missing data (under the assumption of missing completely at random or missing at random). Further as sensitivity analyses, we will perform ‘per protocol’ analyses, including only those participants who finished post-assessment. Feasibility data (% drop-out rate and number of attended training sessions per participant) will be analysed using descriptive statistics. Data distributions of the feasibility questionnaire items will be visually assessed for normality using q-q plots and statistically using the Shapiro-Wilk test.^90 91^

Data analysis will be conducted using IBM SPSS Statistics for Windows (IBM, Armonk, New York, USA), MatLab (The Mathworks, 2016) and R software (https://www.R-project.org).

### Patient and public involvement

We will conduct a brief semistructured interview at the last visit (V12) to assess the patients’ satisfaction with the trial and answer any upcoming questions. The patients were not involved in designing the intervention. All patients will be informed about the study details (eg, the experimental group they participated in) on completion of the study.

## Ethics and dissemination

The study was approved by the ethics committee of the University Medicine of Greifswald ((BB236/20), date of first approval: 5 January 2021) and will be conducted in accordance with the Helsinki Declaration. All data collected will be pseudonymised. Results of the study will be made accessible to scientific researchers and healthcare professionals via publications in peer-reviewed journals and presentations at national and international conferences. Furthermore, the scientific and lay public can access the study results on the ClinicalTrials.gov website (identifier: NCT04817566).

### Protocol amendments

Any substantial amendment to the study protocol will be submitted to the Institutional Ethics Committee for review and approval.

### Confidentiality

The collected data will be treated as confidential. Direct access to personal information and source data documentation will only be given to study monitors, study assessors, and the research team.

## Supplementary material

10.1136/bmjopen-2024-096162 online supplemental file 1

## References

[1] Kaplan HG, Malmgren JA, Atwood MK (2015). Effect of treatment and mammography detection on breast cancer survival over time: 1990-2007. Cancer.

[2] Mounier NM, Abdel-Maged A-S, Wahdan SA (2020). Chemotherapy-induced cognitive impairment (CICI): An overview of etiology and pathogenesis. Life Sci.

[3] Vitali M, Ripamonti CI, Roila F (2017). Cognitive impairment and chemotherapy: a brief overview. Crit Rev Oncol Hematol.

[4] Dijkshoorn ABC, van Stralen HE, Sloots M (2021). Prevalence of cognitive impairment and change in patients with breast cancer: A systematic review of longitudinal studies. Psychooncology.

[5] van der Willik KD, Hauptmann M, Jóźwiak K (2020). Trajectories of Cognitive Function Prior to Cancer Diagnosis: A Population-Based Study. JNCI.

[6] Joly F, Giffard B, Rigal O (2015). Impact of Cancer and Its Treatments on Cognitive Function: Advances in Research From the Paris International Cognition and Cancer Task Force Symposium and Update Since 2012. J Pain Symptom Manage.

[7] Ahles TA, Root JC (2018). Cognitive Effects of Cancer and Cancer Treatments. Annu Rev Clin Psychol.

[8] Collins B, MacKenzie J, Tasca GA (2013). Cognitive effects of chemotherapy in breast cancer patients: a dose-response study. Psychooncology.

[9] Yang Y, Von Ah D (2024). Cancer-related cognitive impairment: updates to treatment, the need for more evidence, and impact on quality of life-a narrative review. Ann Palliat Med.

[10] Von Ah D, Crouch AD, Monahan PO (2022). Association of cognitive impairment and breast cancer survivorship on quality of life in younger breast cancer survivors. J Cancer Surviv.

[11] Han R, Yang YM, Dietrich J (2008). Systemic 5-fluorouracil treatment causes a syndrome of delayed myelin destruction in the central nervous system. J Biol.

[12] Seigers R, Schagen SB, Van Tellingen O (2013). Chemotherapy-related cognitive dysfunction: current animal studies and future directions. Brain Imaging Behav.

[13] Kesler SR, Watson C, Koovakkattu D (2013). Elevated prefrontal myo-inositol and choline following breast cancer chemotherapy. Brain Imaging Behav.

[14] Wefel JS, Kesler SR, Noll KR (2015). Clinical characteristics, pathophysiology, and management of noncentral nervous system cancer‐related cognitive impairment in adults. *CA A Cancer J Clinicians*.

[15] Madhyastha S, Somayaji SN, Rao MS (2002). Hippocampal brain amines in methotrexate-induced learning and memory deficit. Can J Physiol Pharmacol.

[16] Floyd R, Dyer AH, Kennelly SP (2021). Non-pharmacological interventions for cognitive impairment in women with breast cancer post-chemotherapy: A systematic review. J Geriatr Oncol.

[17] Rapp SR, Dressler EV, Brown WM (2024). Phase III Randomized, Placebo-Controlled Clinical Trial of Donepezil for Treatment of Cognitive Impairment in Breast Cancer Survivors After Adjuvant Chemotherapy (WF-97116). JCO.

[18] Mar Fan HG, Clemons M, Xu W (2008). A randomised, placebo-controlled, double-blind trial of the effects of d-methylphenidate on fatigue and cognitive dysfunction in women undergoing adjuvant chemotherapy for breast cancer. *Support Care Cancer*.

[19] Park J-H, Jung SJ, Lee LJ (2023). Impact of nonpharmacological interventions on cognitive impairment in women with breast cancer: A systematic review and meta-analysis. Asia Pac J Oncol Nurs.

[20] Yang P, Hu Q, Zhang L (2025). Effects of non-pharmacological interventions on cancer-related cognitive impairment in patients with breast cancer: A systematic review and network meta-analysis. Eur J Oncol Nurs.

[21] Campbell KL, Zadravec K, Bland KA (2020). The Effect of Exercise on Cancer-Related Cognitive Impairment and Applications for Physical Therapy: Systematic Review of Randomized Controlled Trials. Phys Ther.

[22] Cifu G, Power MC, Shomstein S (2018). Mindfulness-based interventions and cognitive function among breast cancer survivors: a systematic review. BMC Cancer.

[23] Yan X, Wei S, Liu Q (2023). Effect of cognitive training on patients with breast cancer reporting cognitive changes: a systematic review and meta-analysis. BMJ Open.

[24] Garland SN, Savard J, Dalton K (2021). Rationale and protocol for a randomized waitlist controlled trial of videoconference delivered cognitive behaviour therapy for insomnia (CBT-I) to improve perceived cognitive impairment (PCI) among cancer survivors. Contemp Clin Trials.

[25] Derry HM, Jaremka LM, Bennett JM (2015). Yoga and self-reported cognitive problems in breast cancer survivors: a randomized controlled trial. *Psychooncology*.

[26] Wei X, Yuan R, Yang J (2022). Effects of Baduanjin exercise on cognitive function and cancer-related symptoms in women with breast cancer receiving chemotherapy: a randomized controlled trial. Support Care Cancer.

[27] Tong T, Pei C, Chen J (2018). Efficacy of Acupuncture Therapy for Chemotherapy-Related Cognitive Impairment in Breast Cancer Patients. Med Sci Monit.

[28] Vance DE, Frank JS, Bail J (2017). Interventions for Cognitive Deficits in Breast Cancer Survivors Treated With Chemotherapy. Cancer Nurs.

[29] Oldacres L, Hegarty J, O’Regan P (2023). Interventions promoting cognitive function in patients experiencing cancer related cognitive impairment: A systematic review. Psychooncology.

[30] Poppelreuter M, Weis J, Bartsch HH (2009). Effects of specific neuropsychological training programs for breast cancer patients after adjuvant chemotherapy. J Psychosoc Oncol.

[31] Von Ah D, Carpenter JS, Saykin A (2012). Advanced cognitive training for breast cancer survivors: a randomized controlled trial. Breast Cancer Res Treat.

[32] Kesler S, Hadi Hosseini SM, Heckler C (2013). Cognitive training for improving executive function in chemotherapy-treated breast cancer survivors. Clin Breast Cancer.

[33] Park DC, Bischof GN (2013). The aging mind: neuroplasticity in response to cognitive training. Dialogues Clin Neurosci.

[34] Antal A, Alekseichuk I, Bikson M (2017). Low intensity transcranial electric stimulation: Safety, ethical, legal regulatory and application guidelines. Clin Neurophysiol.

[35] Filmer HL, Dux PE, Mattingley JB (2014). Applications of transcranial direct current stimulation for understanding brain function. Trends Neurosci.

[36] Stagg CJ, Nitsche MA (2011). Physiological basis of transcranial direct current stimulation. Neuroscientist.

[37] Brem A-K, Ran K, Pascual-Leone A (2013). Learning and memory. Handb Clin Neurol.

[38] Rodella C, Bernini S, Panzarasa S (2022). A double-blind randomized controlled trial combining cognitive training (CoRe) and neurostimulation (tDCS) in the early stages of cognitive impairment. Aging Clin Exp Res.

[39] Antonenko D, Fromm AE, Thams F (2024). Cognitive training and brain stimulation in patients with cognitive impairment: a randomized controlled trial. *Alz Res Therapy*.

[40] Gonzalez PC, Fong KNK, Brown T (2021). Transcranial direct current stimulation as an adjunct to cognitive training for older adults with mild cognitive impairment: A randomized controlled trial. Ann Phys Rehabil Med.

[41] Pallanti S, Grassi E, Knotkova H (2023). Transcranial direct current stimulation in combination with cognitive training in individuals with mild cognitive impairment: a controlled 3-parallel-arm study. CNS Spectr.

[42] Yang T, Liu W, He J (2024). The cognitive effect of non-invasive brain stimulation combined with cognitive training in Alzheimer’s disease and mild cognitive impairment: a systematic review and meta-analysis. *Alz Res Therapy*.

[43] Chan A-W, Tetzlaff JM, Altman DG (2013). SPIRIT 2013 statement: defining standard protocol items for clinical trials. Ann Intern Med.

[44] Chan A-W, Tetzlaff JM, Gøtzsche PC (2013). SPIRIT 2013 explanation and elaboration: guidance for protocols of clinical trials. BMJ.

[45] Association AP (2013). Diagnostic and statistical manual of mental disorders.

[46] Woods AJ, Antal A, Bikson M (2016). A technical guide to tDCS, and related non-invasive brain stimulation tools. Clin Neurophysiol.

[47] Knecht S, Dräger B, Deppe M (2000). Handedness and hemispheric language dominance in healthy humans. Brain (Bacau).

[48] Lezak MD, Howieson DB, Bigler ED (2012). Neuropsychological Assessment.

[49] Dahlin E, Neely AS, Larsson A (2008). Transfer of learning after updating training mediated by the striatum. Science.

[50] Hartley T, Maguire EA, Spiers HJ (2003). The Well-Worn Route and the Path Less Traveled. Neuron.

[51] Cella D, Choi SW, Condon DM (2019). PROMIS Adult Health Profiles: Efficient Short-Form Measures of Seven Health Domains. Value Health.

[52] Lewis JR (2002). Psychometric Evaluation of the PSSUQ Using Data from Five Years of Usability Studies. Int J Hum Comput Interact.

[53] Johns MW (1991). A New Method for Measuring Daytime Sleepiness: The Epworth Sleepiness Scale. Sleep.

[54] Horne JA, Ostberg O (1976). A self-assessment questionnaire to determine morningness-eveningness in human circadian rhythms. Int J Chronobiol.

[55] Antonenko D, Külzow N, Sousa A (2018). Neuronal and behavioral effects of multi-day brain stimulation and memory training. Neurobiol Aging.

[56] Park S-H, Seo J-H, Kim Y-H (2014). Long-term effects of transcranial direct current stimulation combined with computer-assisted cognitive training in healthy older adults. Neuroreport.

[57] Jones KT, Stephens JA, Alam M (2015). Longitudinal Neurostimulation in Older Adults Improves Working Memory. PLoS One.

[58] Borm GF, Fransen J, Lemmens WAJG (2007). A simple sample size formula for analysis of covariance in randomized clinical trials. J Clin Epidemiol.

[59] Antonenko D, Thams F, Grittner U (2022). Randomized trial of cognitive training and brain stimulation in non-demented older adults. Alzheimers Dement (N Y).

[60] Floel A, Cohen LG (2010). Recovery of function in humans: cortical stimulation and pharmacological treatments after stroke. Neurobiol Dis.

[61] Gandiga PC, Hummel FC, Cohen LG (2006). Transcranial DC stimulation (tDCS): a tool for double-blind sham-controlled clinical studies in brain stimulation. Clin Neurophysiol.

[62] Kuo H-I, Bikson M, Datta A (2013). Comparing cortical plasticity induced by conventional and high-definition 4 × 1 ring tDCS: a neurophysiological study. Brain Stimul.

[63] Schlaug G, Renga V (2008). Transcranial direct current stimulation: a noninvasive tool to facilitate stroke recovery. Expert Rev Med Devices.

[64] Helmstaedter C, Lendt M, Lux S (2001). Verbaler Lern-Und Merkfähigkeitstest: VLMT; manual: Beltz-test.

[65] Stroop JR (1935). Studies of interference in serial verbal reactions. J Exp Psychol.

[66] Strauss E, Sherman EMS, Spreen O (2006). A compendium of Neuropsychological tests: administration, norms, and commentary.

[67] Meyers JE, Meyers KR (1995). Rey complex figure test and recognition trial.

[68] Sweet LH, Kreutzer JS, DeLuca J, Caplan B (2011). Encyclopedia of clinical neuropsychology.

[69] Grabe H, Schulz A, Schmidt C (2012). Ein Screeninginstrument für Missbrauch und Vernachlässigung in der Kindheit: der Childhood Trauma Screener (CTS). Psychiat Prax.

[70] Margraf J, Cwik JC, Suppiger A (2017). DIPS open access: diagnostisches interview bei psychischen Störungen.

[71] Fayers P, Bottomley A (2002). Quality of life research within the EORTC—the EORTC QLQ-C30. Eur J Cancer.

[72] Webster K, Cella D, Yost K (2003). The Functional Assessment of Chronic Illness Therapy (FACIT) Measurement System: properties, applications, and interpretation. Health Qual Life Outcomes.

[73] Buysse DJ, Reynolds CF, Monk TH (1989). The Pittsburgh Sleep Quality Index: a new instrument for psychiatric practice and research. Psychiatry Res.

[74] Windhoff M, Opitz A, Thielscher A (2013). Electric field calculations in brain stimulation based on finite elements: an optimized processing pipeline for the generation and usage of accurate individual head models. Hum Brain Mapp.

[75] Field modeling for transcranial magnetic stimulation: a useful tool to understand the physiological effects of TMS?.

[76] Antonenko D, Hayek D, Netzband J (2019). tDCS-induced episodic memory enhancement and its association with functional network coupling in older adults. Sci Rep.

[77] Darki F, Klingberg T (2015). The role of fronto-parietal and fronto-striatal networks in the development of working memory: a longitudinal study. Cereb Cortex.

[78] Whitfield-Gabrieli S, Nieto-Castanon A (2012). Conn: a functional connectivity toolbox for correlated and anticorrelated brain networks. Brain Connect.

[79] Yendiki A, Panneck P, Srinivasan P (2011). Automated probabilistic reconstruction of white-matter pathways in health and disease using an atlas of the underlying anatomy. Front Neuroinform.

[80] Le Bihan D, Johansen-Berg H (2012). Diffusion MRI at 25: Exploring brain tissue structure and function. Neuroimage.

[81] Metzler-Baddeley C, Jones DK, Belaroussi B (2011). Frontotemporal Connections in Episodic Memory and Aging: A Diffusion MRI Tractography Study. J Neurosci.

[82] Metzler-Baddeley C, Foley S, de Santis S (2017). Dynamics of White Matter Plasticity Underlying Working Memory Training: Multimodal Evidence from Diffusion MRI and Relaxometry. J Cogn Neurosci.

[83] Charlton RA, Barrick TR, Lawes INC (2010). White matter pathways associated with working memory in normal aging. Cortex.

[84] Jenkinson M, Beckmann CF, Behrens TEJ (2012). FSL. Neuroimage.

[85] Smith SM, Jenkinson M, Woolrich MW (2004). Advances in functional and structural MR image analysis and implementation as FSL. Neuroimage.

[86] Brodt S, Gais S, Beck J (2018). Fast track to the neocortex: A memory engram in the posterior parietal cortex. Science.

[87] Filmer HL, Ehrhardt SE, Shaw TB (2019). The efficacy of transcranial direct current stimulation to prefrontal areas is related to underlying cortical morphology. Neuroimage.

[88] Guarienti F, Caumo W, Shiozawa P (2015). Reducing transcranial direct current stimulation-induced erythema with skin pretreatment: considerations for sham-controlled clinical trials. *Neuromodulation*.

[89] McFadden JL, Borckardt JJ, George MS (2011). Reducing procedural pain and discomfort associated with transcranial direct current stimulation. Brain Stimul.

[90] Rahbek MA, Mikkelsen EE, Overgaard K (2017). Exercise in myasthenia gravis: A feasibility study of aerobic and resistance training. Muscle Nerve.

[91] Bock BC, Thind H, Fava JL (2019). Feasibility of yoga as a complementary therapy for patients with type 2 diabetes: The Healthy Active and in Control (HA1C) study. Complement Ther Med.

[92] Passow S, Thurm F, Li S-C (2017). Activating Developmental Reserve Capacity Via Cognitive Training or Non-invasive Brain Stimulation: Potentials for Promoting Fronto-Parietal and Hippocampal-Striatal Network Functions in Old Age. Front Aging Neurosci.

[93] Watson D, Clark LA, Tellegen A (1988). Development and validation of brief measures of positive and negative affect: The PANAS scales. J Pers Soc Psychol.

