# Supplementary material for: Neuromodulation through brain stimulation-assisted cognitive training in patients with post-chemotherapy subjective cognitive impairment (Neuromod-PCSCI) after breast cancer: study protocol for a double-blinded randomised controlled trial
Source: BMJ Open. 2025 May 21;15(5):e096162. doi: 10.1136/bmjopen-2024-096162 (PMC12096976; doi:10.1136/bmjopen-2024-096162)
Supplement: online supplemental file 1 [file bmjopen-15-5-s001.docx]

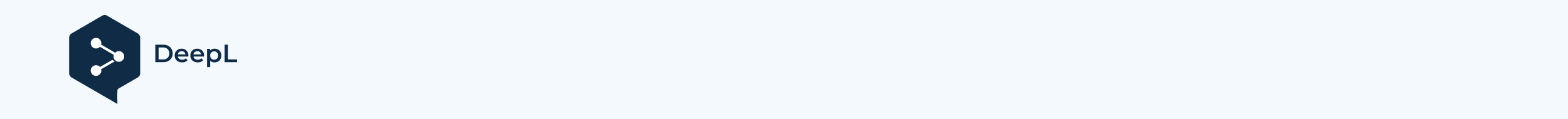


Subscribe to DeepL Pro to edit this document.
Visit [www.DeepL.com/pro](https://www.deepl.com/pro?cta=edit-document) for more information.

**
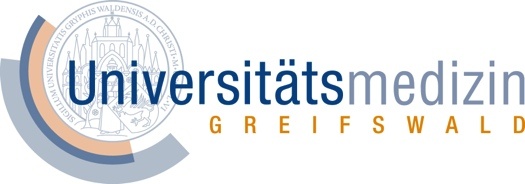
**

**Participant information for the study**

**"Neuromodulation through brain stimulation and cognitive training in female cancer patients with chemotherapy-induced cognitive impairment (Neuromod-PCCI)"**

Dear study participant,

Thank you for your interest in the above-mentioned study. This study is a basic scientific research project designed to investigate the influence of cognitive training in combination with non-invasive brain stimulation on your performance and on the transmission of information in your brain. As required by law, our study has been approved by the relevant ethics committee.

The study methods, expected benefits and possible risks of this study are described below. Please take sufficient time to decide whether you would like to participate and do not hesitate to raise any points that are unclear to you. The principal investigators and study physicians are always available to answer your questions.

**Study management**

Prof. Dr. med. Agnes Flöel

Ferdinand-Sauerbruch-Straße

17475 Greifswald

Phone: +49 3834 86-6815

Email: agnes.floeel@med.uni-greifswald.de

1. **Background and aim of the study**

A large proportion of breast cancer patients develop cognitive impairments after chemotherapy. These impairments can last for years after the end of chemotherapy and occur in various areas such as memory, cognitive control, processing speed and attention. An interesting new approach to improving such abilities is to simultaneously stimulate brain activity during task performance. This so-called transcranial electrical stimulation is achieved by attaching electrodes to the surface of the head from the outside, through which a weak, barely perceptible current is administered. This can change brain activity in superficial layers of the brain.
The aim of this study is to test the effectiveness of such stimulation in combination with intensive cognitive control training (lasting several days).

1. **Procedure of the study**

This study includes several appointments at the University Medical Center Greifswald, during which we will carry out various examinations with you. You will attend a total of thirteen appointments.

During the initial examination, you will be given detailed written and verbal information about the individual examinations (procedure, risks) and have the opportunity to clarify any questions with the examiner. You will also undergo a detailed neuropsychological examination. This will clarify whether you can take part in the study without any health risks. If there are no concerns, you can decide whether you would like to take part in the study voluntarily. You can withdraw your consent at any time. Of course, the results will not be passed on to third parties and medical confidentiality is guaranteed (for further data protection information, see consent form). You will receive detailed instructions from us during the examination. The initial examinations begin with a few standard questions about your health, general information about yourself and how you feel. As part of the initial examinations, blood is taken from a vein in your arm to examine certain polymorphisms (different manifestations in genes) in genes relevant to learning, as well as to examine inflammatory processes in the body. This is followed by a diagnostic interview and a series of standardized psychological tests to assess various performance-related characteristics, such as attention or memory performance

Following the initial examination, you will be invited to nine appointments (three times a week for three consecutive weeks). At all appointments, electrodes are attached to your head using a cap, through which the stimulation is applied. This involves either "real" anodal direct current stimulation or so-called sham (or placebo) stimulation. The allocation to these two groups is random and is not known to you or the study leader who is conducting the test with you. At the same time as the stimulation, you will be asked to complete experimental cognitive tasks on a tablet. The handling of the devices and the experimental task will be explained to you in detail in advance so that all your questions can be discussed. The instructions for the tasks will also appear on the screen immediately before you start.

In order to measure the effects of the cognitive training, you will be invited to two follow-up examinations, one immediately after the training period and one approximately four weeks later.

1. **What are the personal benefits of participating in the study**?

By participating in this study, you will receive a detailed personal performance assessment of your cognitive abilities. Through the intensive practice of cognitive control functions, we expect an improvement in the trained functions in all participants, which may also be recognizable in other tasks. The results of the study may help to improve the treatment of cognitive disorders after chemotherapy. This would be an important prerequisite for providing patients with better advice in the future and possibly even developing preventive measures

1. **What are the risks associated with participating in the study?**

Overall, the risk of possible damage to health from the examination is considered to be minimal. Possible contraindications, i.e. circumstances that contradict the use of stimulation, for example, are clarified by means of a detailed initial examination by experienced personnel.

Non-invasive electrical brain stimulation

*What is stimulation?* Transcranial electrical stimulation is a technique that makes it possible to painlessly generate a current flow in the brain from the scalp and thus influence the excitability of the brain. This is done using a battery-operated stimulator and five surface electrodes.

At the start of each appointment, the electrodes are attached to the surface of your head using a cap. A small external current is applied via the electrodes, which may initially cause a brief tingling sensation on the scalp. In the sham stimulation, the stimulation is stopped after a few seconds - imperceptibly for you - and only continued in the real stimulation.

*What side effects are to be feared?* With the stimulation parameters used here (20 min stimulation with 2.0 mA per intervention), no side effects have occurred to date apart from a tingling sensation under the electrodes. Transcranial electrical stimulation has already been used in several clinical studies and has proven to be low-risk and with few side effects. Current intensities and electrode sizes are used that lead to a current flow below the perception threshold. Studies have so far only reported after-effects in the form of slight itching of the skin under the electrodes during the current flow and flickering of light when the current is switched on and off. All effects are reversible. Rarely and only after continuous electrical stimulation for hours did slight headaches and skin irritation occur in the area of the electrodes, which quickly subsided completely.

Venous blood sampling for genetic testing:

Venous blood sampling is performed by experienced staff after prior disinfection of the puncture site. Differences in response to the stimulation carried out here can possibly be explained in part by different manifestations in genes (genetic polymorphisms) that are important for learning, as well as by pre-existing inflammatory reactions in the body (partly triggered by chemotherapy). The blood taken will be analyzed exclusively with regard to such learning-relevant genetic polymorphisms and possible inflammatory processes. Only study-related data will be obtained. Since it is only a minimal part of the DNA information that is analyzed, no information about genetic diseases can be obtained. The blood taken is not identified by name, but is given a code (pseudonymized) so that no conclusions can be drawn about your person in the analysis laboratory. Only the study team has access to the code that allows the study-related data to be linked to you. Possible risks such as complications like hematomas, infection or pain at the injection site are possible, but rare. These complications are easily treatable and completely reversible

Local anesthesia:

*What is EMLA cream?* Before stimulation, a superficial anaesthetic cream is applied to the electrode sites to slightly numb the sensations on the scalp. EMLA-Creme® (25 mg/g lidocaine + 25 mg/g prilocaine) is applied to your scalp for this purpose. After a contact time of about 20 minutes, the cream is removed and we begin with the stimulation build-up.

*What side effects are to be feared?* After we have applied EMLA-Creme® externally to the skin, the two active ingredients are distributed in this area and block the pain stimulus conduction for a certain period of time. This can cause discomfort in this area. In addition, the skin in these areas may feel warm, itchy and slightly reddened. Very rarely and after prolonged use, the concentration of methaemoglobin (a version of the blood pigment) may become too high in the case of certain pre-existing conditions. As EMLA-Creme® is applied superficially and does not come into contact with the blood, the likelihood of such a side effect developing is very low. We will discuss everything that speaks against the use of EMLA-Cream® with you thoroughly beforehand.

1. **Who can take part in this study?**

You can only take part in this study if you are female, between 18 and 65 years of age and have received chemotherapy for the treatment of breast cancer six months or more ago. If you have no neurological or psychiatric conditions other than self-reported cognitive impairment, and you are not simultaneously volunteering for other studies or other clinical research projects, you may participate in the study. Patients with severe neurological disorders, a history of severe alcoholism or drug and medication abuse, and severe psychiatric disorders such as depression (except in remission), psychosis and severe untreated medical problems will be excluded from participation.

1. **Allowance for expenses**

Depending on the appointment, the examination lasts from approx. 45 minutes to two hours. For your cooperation in the study, you will receive an expense allowance of €10 per hour after completion of all examinations. If you complete all 13 appointments, this corresponds to compensation of €180. If we have to terminate the study, you will receive a pro rata expense allowance.

1. **Am I insured during the study?**

A commuting accident insurance policy has been taken out for you (insurer: SV SparkassenVersicherungs AG, insurance number: 50090011284). We will provide you with a copy of the insurance conditions and further information.

1. **Voluntary nature of participation**

Participation in the study is voluntary; you can withdraw your consent at any time and without giving reasons, without this resulting in any disadvantages for you. If you withdraw your consent to participate in the study, you have the right to request the deletion of all your data collected up to that point.

1. **Data protection**

All data collected in the course of the study is treated in strict confidence. The data is stored and evaluated pseudonymously and without a personal identifier. Pseudonymized means that no names or initials are used, only a number and/or letter code. The data is protected against unauthorized access. The existing pseudonymized data is stored for a period of 10 years in accordance with good scientific practice. You can object to the further processing of your data at any time and request its deletion

The blood taken for this test is pseudonymized and examined for certain cognition-related genes in the laboratory of the Faculty of Pharmacy under the direction of Prof. Tzvetkov. Only a minimal part of the DNA is analyzed, from which no information about genetic diseases can be obtained. The investigation of possible inflammatory processes is carried out pseudonymously in the research laboratory MVZ Labor Krone GbR in Bad Salzulfen. Naturally, the results are not passed on to third parties and medical confidentiality is guaranteed.

As part of this study, we are working with the German PROMIS National Center (GPNC) under the direction of our cooperation partner Prof. Sein Schmidt. We transmit your pseudonymized personal data (PROMIS data records, data about your social, economic and professional background, as well as information about your cancer) to the GPNC for recording in a reference database. You can also object to this further processing of your data at any time.

1. **Who can I contact if I have any questions about the study?**

If you have any questions, please contact the study coordinators Prof. Agnes Flöel and Dr. Daria Antonenko (see above for e-mail addresses and telephone numbers).

**Declaration of consent for the study**

**"Neuromodulation through brain stimulation and cognitive training in female cancer patients with chemotherapy-induced cognitive impairment (Neuromod-PCCI)"**

Name of the test person in block capitals_____________________________________

Date of birth: _____________ (DD/MM/YYYY)

Participant number: _____________ (to be filled in by the person responsible for the study)

I hereby declare that I have been informed verbally and in writing by Ms/Mr. _____________________________ about the study and the associated objectives, the procedure and possible risks. I have understood the patient information in all details, I have had sufficient time to think about it and any questions that arose have been adequately answered. I am aware that participation in the study can be terminated at any time without giving reasons and without any disadvantages for me. I voluntarily agree to participate in the above-mentioned study. I will receive a written version of the patient information and declaration of consent after I have signed it.

**Data protection:**

I am aware that personal data will be collected, stored and analyzed in this study. The use of my health data is in accordance with legal regulations and requires the following voluntary declaration of consent prior to participation in the study, i.e. without the following consent I cannot participate in the study.

1. I agree that personal data collected in the context of this study will be recorded and processed pseudonymized (encrypted) in paper form and on electronic data carriers. I agree that my pseudonymized personal data (PROMIS data sets, sociodemographic core data, as well as primary disease entity) may be transmitted to the German PROMIS National Center (GPNC) under the direction of our cooperation partner Prof. Sein Schmidt in order to be recorded there within the normative PROMIS reference database.

The person responsible for data processing in the study is Professor Dr. med. Agnes Flöel (Clinic and Polyclinic for Neurology, Ferdinand-Sauerbruch-Straße, 17475 Greifswald, phone: 03834-86-6815, e-mail: agnes.floeel@uni-greifswald.de).

is the data protection officer:

Prof. Ulf Glende

Group Data Protection Officer

University Medicine Greifswald

Walther-Rathenau-Str. 49 * 17489 Greifswald

Phone: +49 3834 86-5124

E-Mail: [datenschutz-umg@med.uni-greifswald.de](mailto:datenschutz-umg@med.uni-greifswald.de)

I have been made aware of the right to lodge a complaint with a data protection supervisory authority. The data protection supervisory authority responsible for the study is: Der Landesbeauftragte für Datenschutz und Informationsfreiheit Mecklenburg-Vorpommern, Werderstraße 74a, 19055 Schwerin, E-Mail: [info@datenschutz-mv.de](mailto:info@datenschutz-mv.de)

2. I have been informed that I can withdraw my participation in the study at any time and without giving reasons. If I withdraw my consent, I have the right to object to the further processing of my data collected as part of the above-mentioned study and to request its deletion or to agree that the data stored up to this point in time - in anonymized form - may continue to be used.

3. I agree that my data will be stored for at least ten years after completion or discontinuation of the study, as stipulated by the regulations on studies. After this period, my personal data will be deleted, unless there are legal, statutory or contractual retention periods to the contrary.

4. I have taken note of my right to inspect and correct my personal data under data protection law. I have been informed of the right to obtain information about personal data concerning me and, if necessary, to request its correction or deletion.

5. Finally, I also give my consent for the scientific publication of the research results in compliance with data protection regulations. The data will only be published in anonymized form.

**I agree to participate voluntarily in the above-mentioned study.**

I have received a copy of the subject information and consent form. One copy remains at the study site.

...........................................................................................................................

Name of the test person in block capitals

.................................... ...............................................................................

Date Signature of the **test person**

I conducted the informed consent interview and obtained the subject's consent.

...........................................................................................................................

Name of the director of studies

.................................... ...............................................................................

Date Signature of the informing **study director/study doctor**
