# Supplementary material for: Neuromodulation through brain stimulation-assisted cognitive training in patients with post-chemotherapy subjective cognitive impairment (Neuromod-PCSCI) after breast cancer: study protocol for a double-blinded randomised controlled trial
Source: BMJ Open. 2025 May 21;15(5):e096162. doi: 10.1136/bmjopen-2024-096162 (PMC12096976; doi:10.1136/bmjopen-2024-096162)
Supplement: Supplementary file 2 [file bmjopen-2024-096162.reviewer_comments.pdf]

PEER REVIEW HISTORY

BMJ Open publishes all reviews undertaken for accepted manuscripts. Reviewers are asked to complete a checklist review form and are provided with free text boxes to elaborate on their assessment. These free text comments are reproduced below.

ARTICLE DETAILS

Title (Provisional)

Neuromodulation through Brain Stimulation-assisted Cognitive Training in Patients with Post-Chemotherapy Subjective Cognitive Impairment (Neuromod-PCSCI) after Breast Cancer – Study Protocol of a Double-Blinded Randomized Controlled Trial

Authors

Rocke, Merle; Knochenhauer, Elena; Thams, Friederike; Antonenko, Daria; Fromm, Anna Elisabeth; Jansen, Nora; Aziziaram, Samaneh; Grittner, Ulrike; Schmidt, Sein; Vogelgesang, Antje; Brakemeier, Eva-Lotta; Flöel, Agnes

---

VERSION 1 - REVIEW

**Reviewer**	**1**
**Name**	**Cáceres, Macarena C.**
**Affiliation**	**Universidad de Extremadura**
**Date**	**24-Jan-2025**
**COI**	**None**

General comments

This is a very relevant study given the importance of chemotherapy-induced cognitive impairment in women with breast cancer. It is a topic that is largely unaddressed in clinical practice and this should be addressed.

Specific comments

Tittle:

The title should include the word breast cancer.

Introduction:

More data from recent studies on non-pharmacological interventions to treat breast cancer-related cognitive impairment should be included.

The objective of the clinical trial should be more clearly stated in the introduction or in a specific section for this purpose.

Methods:

I have doubts about the structure of the Methods section. The subheadings that are part of this section do not really reflect everything they contain, so it might be worth considering changing their name or deleting them altogether. The section 'Methods: Assignment of Interventions' only contains the sub-section on 'Blinding', so perhaps it should read 'Randomization and Blinding'.

With regard to the timeline, although reference is made to the participant timeline, the dates of the study are not included.

A more detailed description of the frequency and methods of data auditing is missing.

It should be explained why only right-handed people will be included in the study.

The battery of questionnaires is very complete, but I wonder if they have considered including the FACT Cog, as it is one of the most widely used tools in this type of study.

Conclusions:

The wording of the conclusions should be reconsidered. Only reference is made to the working memory, why?

References:

Although it is logical that the topicality of the bibliographical references in a protocol cannot be completely controlled, as they mainly refer to materials and methods, I do believe that an effort should be made to include more current references in the introduction section.

**Reviewer**	**2**
**Name**	**Araujo, Natalia**
**Affiliation**	**University of Porto Epidemiology Research Unit**
**Date**	**05-Mar-2025**
**COI**	**None**

This manuscript describes a very interesting intervention to improve working memory and, as secondary objectives, to improve other cognitive domains and health-related quality of life in women treated with chemotherapy at least six months ago, for breast cancer and who have concerns about cognitive function.

In general, the structure of the manuscript could be improved to reduce the repetition of information.

Indeed, the information presented in sections "Baseline Measures" and "Pre., Post-, and Follow-up assessments" are further repeated in sub-sections of the section "Methods: Data Collection, Management, and Analysis." For example, questionnaires and tests are listed on page 10 and again on page 12; similarly, quality-of-life questionnaires are presented on page 10 and then described on pages 13-14.

A figure with the timeline of procedures would improve the understanding of the study design, namely on which days each task occurs. Indeed, nine sessions of the interventions are planned for three consecutive weeks but in which days will the interventions be administered? D1, D2, D3, then, D8, D10, D11, then, D15, D16, D17 or D1, D3, D5, D8, D10, D12, D15, D17, D19, or any other sequence?

Important aspects: how is this intervention specifically for post-chemotherapy cognitive impairment (PCCI)? If there is no objective identification of PCCI, then the intervention aims to improve working memory, and the performance in other cognitive domains and health-related quality of life of women previously treated with chemotherapy for breast cancer, not women with PCCI.

Indeed:

1) The authors refer “post-chemotherapy cognitive impairment” but the inclusion criteria include “Self-reported concerns regarding cognitive function”, which is common to many people. Inclusion criteria do not include cognitive impairment, either clinically relevant subjective complains or objectively assessed cognitive impairment;

2) There is no description of how is “post-chemotherapy” assessed: will participants give information on the existence of cognitive complains before chemotherapy and breast cancer diagnosis?

With this regard, please consider adding in the introduction, that many studies have presented the occurrence of cognitive impairment (in women with breast cancer and other cancers) before cancer treatment and after treatments others than chemotherapy.

Eligibility criteria lead to a very heterogeneous study population: how long after breast cancer chemotherapy? Is the intervention designed to be administered to any women who had/has breast cancer and was treated with chemotherapy in the past, even 20 years ago? Will women who had previously another cancer or who develop another cancer after chemotherapy for breast cancer be included?

With such a heterogeneous population, the small sample size of 52 participants will probably be not enough to warranty two groups of participants with similar characteristics, namely those considered relevant for cognitive outcomes “education, baseline cognitive ability, neuropsychological status as well as socio-demographic and clinical characteristics” (pages 8-9). The authors should discuss this issue/include this limitation in the bullet points, or increase sample size. Additionally, they should explain why they decided to stratify the selection of participants based on a specific cut-off of 87% in the baseline n-back task.

Please clarify “double blinded”: will participants and all members of the research team, except who administer the intervention, be blinded?

“Double-blinded” should be in the title.

Define “excessive consumption of alcohol or nicotine on the days of intervention”.

Page 9, briefly present the n-back task and give references when first referred.

VERSION 1 - AUTHOR RESPONSE

Reviewer #1

Dr. Macarena C. Cáceres, Universidad de Extremadura

Comments to the Author:

General comments

This is a very relevant study given the importance of chemotherapy-induced cognitive impairment in women with breast cancer. It is a topic that is largely unaddressed in clinical practice and this should be addressed.

We sincerely thank Dr. Macarena C. Cáceres for her insightful comments and constructive suggestions. We believe these revisions have significantly improved the clarity of the Methods section and enhanced the Introduction by incorporating the latest literature on non-pharmacological interventions for PCCI. Our detailed responses are listed below.

Specific comments

Tittle:

The title should include the word breast cancer.

We thank the reviewer for the suggestion. Based on overall suggestions from reviewer 1 and 2 the title now reads:

Neuromodulation through Brain Stimulation-assisted Cognitive Training in Patients with Post-Chemotherapy Subjective Cognitive Impairment (Neuromod-PCSCI) after Breast Cancer – Study Protocol of a Double-Blinded Randomized Controlled Trial

The term Neuromod-PCSCI will replace our former term Neuromod-PCCI throughout the whole manuscript.

Introduction:

More data from recent studies on non-pharmacological interventions to treat breast cancer-related cognitive impairment should be included.

We agree with the reviewer that incorporating the latest literature on non-pharmacological interventions for PCCI will significantly enhance the introduction. It now reads for example on page 4:

Thus far, no evidence-based pharmacological treatments are available for post-chemotherapy cognitive impairment (PCCI)[1]. Although agents like donepezil[2] and methylphenidate[3] have been studied in randomized controlled trials, these studies have not shown significant improvements in cognitive function compared to a placebo, and neither of these agents has been approved. Due to potential side effects and the lack of robust evidence for pharmacological means, non-pharmacological interventions have gained increasing attention and popularity.

Many studies have shown that non-pharmacological interventions such as cognitive rehabilitation, physical activities, mindfulness therapies, cognitive-behavioral therapy, and

acupuncture can be effective in improving cancer-related cognitive impairment in breast cancer survivors[1 4 5]. Meta-analyses and systematic reviews have reported beneficial effects of physical exercise[6], mindfulness-based interventions[7], and particularly cognitive training[8] on both subjective cognitive complaints and objective cognitive performance. Cognitive behavioral therapy has demonstrated efficacy in alleviating psychological distress and improving self-perceived cognitive functioning[9], while interventions such as yoga and aerobic exercise have shown additional emotional and cognitive benefits[10 11]. Acupuncture has also been investigated as a complementary method, with promising results[12].

Vance et al.[13] demonstrated that cognitive training interventions and compensatory strategies led to improvements in cognitive deficits among breast cancer survivors who had received chemotherapy. Similarly, a systematic review by Oldacres et al.[14] emphasized that cognitive training is among the most widely studied and promising interventions for cancer-related cognitive impairment.

In a meta-analysis by Yan et al.,[8] nine randomized controlled trials (RCTs) involving a total of 666 patients with breast cancer were reviewed to assess the effectiveness of cognitive training in patients with cognitive changes. The evidence suggests that cognitive training may improve subjective cognitive function, processing speed, verbal memory, working memory, and episodic memory in patients with breast cancer who are reporting cognitive changes.

The objective of the clinical trial should be more clearly stated in the introduction or in a specific section for this purpose.

We thank the reviewer for the comment. We have now clarified the objective of the clinical trial at the end of the introduction. It now reads on page 5:

The intervention will span three weeks, with three training sessions per week. We will assess behavioral outcome measures, such as immediate training effects, feasibility, safety and tolerability, transfer to untrained domains, and long-term effects on cognition and quality of life at multiple time points. We will further elucidate neural characteristics associated with interventional success before the intervention and assess neural correlates at post-intervention. This protocol, describing the design and methods of the Neuromod-PCSCI study, was prepared in accordance with the SPIRIT guidelines. With regard to both feasibility and behavioral outcomes, the purpose of the present study is to inform planning and guide protocol development (including power analysis) in relation to future, confirmatory randomized controlled trials (RCTs) on the efficacy of cognitive training-plus-tDCS in increasing cognitive functions in patients with PCCI

Methods:

I have doubts about the structure of the Methods section. The subheadings that are part of this section do not really reflect everything they contain, so it might be worth considering changing their name or deleting them altogether. The section 'Methods: Assignment of Interventions' only contains the sub-section on 'Blinding', so perhaps it should read 'Randomization and Blinding'.

We thank the reviewer for the suggestion. We now changed the name of the section "Methods: Assignment of Interventions" to "Methods: Randomization and Blinding" to clarify the content.

With regard to the timeline, although reference is made to the participant timeline, the dates of the study are not included.

We thank the reviewer for the suggestion and included more comprehensive figure of the training intervention. We will add the Figure for a better understanding of the Table 1, where we listed the outcome measures in combination with the Visits (V0-V12). It now reads on page 9 and 22:

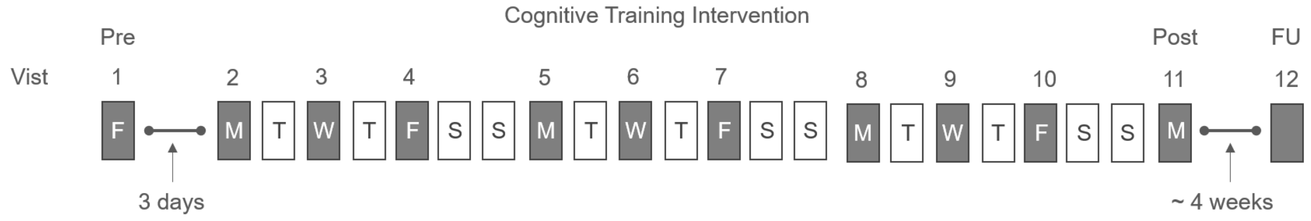

Participant Timeline: Individuals will participate in 13 visits, and two optional MRI sessions, taking place at the University Medicine Greifswald. After inclusion at the baseline visit (V0), participants will attend the pre-assessment visit (on a Friday, V1) before commencing the nine training visits, which will take place over three consecutive weeks, three days a week (V2-V10). The cognitive training intervention will take place on Monday (M), Wednesday (W), and Friday (F). After the training, a post-assessment (on a Monday, V11) will be conducted, and four weeks later, a follow-up visit (V12) will be administered. MRI will be acquired before pre-assessment (V1) and at post-assessment (V13). Figure X provides a concise overview of this participant timeline.

A more detailed description of the frequency and methods of data auditing is missing.

We thank the reviewer for highlighting the need for clearer data auditing details. In addition to our described data security measures, we implemented systematic quality control: (1) weekly automated checks for outliers/missing values in key outcomes, and (2) 100% cross-checking of critical documents (consent forms, adverse events). Protocol adherence is further monitored through equipment logs and weekly team meetings. These procedures have now been added to the Methods. It now reads on page 16:

All participant data will be pseudonymized. Spreadsheets containing both participant IDs and personal data will be protected with a password only known to the study staff and will be stored on a secure file server. Similarly, digital data, i.e., output files from computer-based tasks, will be saved on a secure file server directly after acquisition. Non-digitally acquired data will be manually digitized by a staff member of the research team and double-checked by another member. Progress of data entry and checking procedures will be documented. As additional quality control, we implemented systematic quality control by checking for outliers or missing values in key outcomes on a weekly basis, and cross-checking 100% of critical documents (consent forms, adverse events). Additionally, protocol adherence is further monitored through equipment logs and weekly team meetings. Files containing subject records will be sorted by participant ID and stored securely. Sensitive data, such as names and medical records, will be scored separately in lockable cabinets in rooms with access restricted to the researchers. Protocols of the tDCS setup of each participant and session will also be stored on the file server and MRI data will be pseudonymized before analysis. Following good scientific practice, data will be stored for at least 10 years.

It should be explained why only right-handed people will be included in the study.

We thank the reviewer for the question and the opportunity to validate our inclusion and exclusion criteria.

We restricted participation to right-handed individuals due to well-documented hemispheric differences in cognitive processing and tDCS effects[15-18]. The left dorsolateral prefrontal cortex (DLPFC), our target stimulation site, shows more consistent functional organization and localization in right-handed individuals. By including only right-handed participants, it may be possible to control for potential variability in tDCS response due to handedness-related neuroanatomical differences[18 19]. We additionally avoid confounding effects from potential interhemispheric reorganization in left-handed or ambidextrous participants. This approach follows established tDCS methodology in cognitive studies (e.g., as in[15]) and helps ensure more interpretable results regarding the role of the left DLPFC in post chemotherapy-related deficits.

We added this information to the manuscript. It now reads on page 8:

Participants include right-handed women (18-65 years) with chemotherapy-treated breast cancer (completed ≥6 month prior), excluding those with other cancers, current malignancies, or pre-treatment cognitive complaints. This window captures both subacute and chronic post-chemotherapy effects while controlling for major confounders through strict neurological/psychiatric exclusions. We restricted participation to right-handed individuals due to well-documented hemispheric differences in cognitive processing and tDCS response and our stimulation target exhibits more consistent functional organization in right-handed individuals [15 19], reducing variability in neuromodulation effects. This approach also minimizes confounds from interhemispheric reorganization patterns more common in non-right-handed populations

The battery of questionnaires is very complete, but I wonder if they have considered including the FACT Cog, as it is one of the most widely used tools in this type of study.

Our primary quality-of-life measure is the PROMIS (Patient-Reported Outcomes Measurement Information System, https://www.promishealth.org/57461-2/ in collaboration with Prof. Schmidt). We agree that the FACT-Cog may provide important functional insights. In response to this comment, we will now implement the FACT-Cog for all current and future participants (approximately 30 subjects to date). This addition will allow for a more comprehensive assessment of cognitive-related quality of life while maintaining compatibility with our existing PROMIS data.

This information has now been added to the Methods part (for example page 14:

“• FACT Cog[20], to gain additional insights on perceived cognitive impairments in cancer patients with chemotherapy-induced cognitive problems”

Conclusions:

The wording of the conclusions should be reconsidered. Only reference is made to the working memory, why?

We thank the reviewer for this observation. In the original submission, we focused on working memory outcomes as they were the primary endpoint in our previous trials. However, in response to editorial feedback, we have removed the Conclusions section entirely to maintain consistency with journal guidelines for study protocols. The discussion of all cognitive outcomes, including working memory and other domains, now appears exclusively in the Results and Discussion sections.

References:

Although it is logical that the topicality of the bibliographical references in a protocol cannot be completely controlled, as they mainly refer to materials and methods, I do believe that an effort should be made to include more current references in the introduction section.

We thank the reviewer for this suggestion and have updated the Introduction with the latest literature on non-pharmacological post-chemotherapy cognitive impairment interventions, including recent references.

Reviewer #2

Dr. Natalia Araujo, University of Porto Epidemiology Research Unit

This manuscript describes a very interesting intervention to improve working memory and, as secondary objectives, to improve other cognitive domains and health-related quality of life in women treated with chemotherapy at least six months ago, for breast cancer and who have concerns about cognitive function.
In general, the structure of the manuscript could be improved to reduce the repetition of information.

We sincerely thank Dr. Natalia Araujo for her insightful comments and constructive suggestions. Based on your thoughtful feedback, we are confident that we have improved our manuscript. We have significantly refined the Introduction by eliminating redundant information and improving flow. We also added a more comprehensive definition and discussion of post-chemotherapy cognitive impairment (PCCI) in the updated manuscript

Our detailed responses are listed below.

Indeed, the information presented in sections “Baseline Measures” and “Pre., Post-, and Follow-up assessments” are further repeated in sub-sections of the section “Methods: Data Collection, Management, and Analysis.” For example, questionnaires and tests are listed on page 10 and again on page 12; similarly, quality-of-life questionnaires are presented on page 10 and then described on pages 13-14.

We thank the reviewer for pointing this out. We now summarized the information comprehensively. It now reads on page 9-10:

Baseline Measures: At baseline, participants will provide written informed consent and participate in a demographic interview. The participants will be asked to provide a record of their cancer specific medical history. If study-relevant information is missing, it will be requested from the responsible physician. Furthermore, a diagnostic interview to screen for mental disorders will be conducted (see section: Psychological Assessment). Subsequently the participants will be tested with a comprehensive battery of neuropsychological tests to quantify cognitive function on different domains (see section: Neuropsychological Assessment). For more details, please refer to the following corresponding subsection.

Afterwards, participants will perform the training task as described above […]

Pre-, Post-, and Follow-Up-Assessments: All three sessions will follow the same procedure. First, self-reported well-being, quality, and duration of sleep as well as potential stressors up to two hours prior to the visit will be assessed by the investigator via a questionnaire. The participants will further complete an array of QoL-related questionnaires along with sleep questionnaires (see section: Psychological Assessment). Then, participants will complete the training task and the untrained tasks. The follow-up assessments will provide the possibility for assessing the maintenance of training and transfer effects.

A figure with the timeline of procedures would improve the understanding of the study design, namely on which days each task occurs. Indeed, nine sessions of the interventions are planned for three consecutive weeks but in which days will the interventions be

administered? D1, D2, D3, then, D8, D10, D11, then, D15, D16, D17 or D1, D3, D5, D8, D10, D12, D15, D17, D19, or any other sequence?

We thank the reviewer for the suggestion and included a more comprehensive figure of the training intervention. We will add a figure to enhance the understanding of Table 1, which lists the outcome measures in conjunction with the visits. It now reads on page 9 and 22:

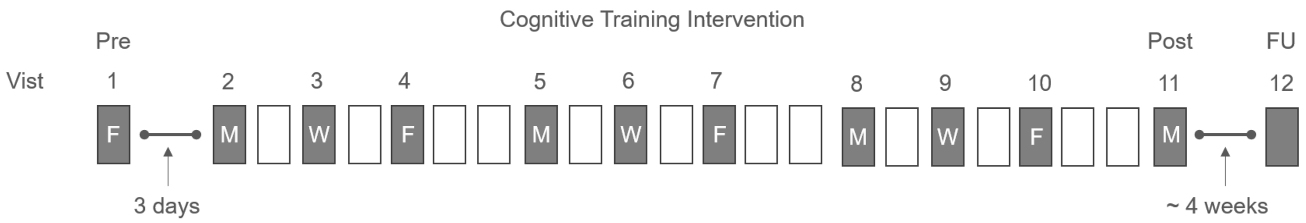

Participant Timeline: Individuals will participate in 13 visits, and two optional MRI sessions, taking place at the University Medicine Greifswald. After inclusion at baseline visit (V0), participants will attend the pre-assessment visit (Friday, V1) before starting the nine training visits during three consecutive weeks on three days a week (V2-V10). The cognitive training intervention will take place on Monday, Wednesday and Friday. After the training, a post-assessment (Monday, V11) will be conducted and four weeks later a follow-up visit (V12) will be administered. MRI will be acquired before pre-assessment (V1) and at post-assessment (V13). Figure 2 provides a concise overview of the participant time line.

Important aspects: how is this intervention specifically for post-chemotherapy cognitive impairment (PCCI)? If there is no objective identification of PCCI, then the intervention aims to improve working memory, and the performance in other cognitive domains and health-related quality of life of women previously treated with chemotherapy for breast cancer, not women with PCCI.

We thank the reviewer for this important methodological question regarding PCCI identification. Below, we have now clarified the study rationale and our inclusion criteria.

Indeed:

1) The authors refer “post-chemotherapy cognitive impairment” but the inclusion criteria include “Self-reported concerns regarding cognitive function”, which is common to many people. Inclusion criteria do not include cognitive impairment, either clinically relevant subjective complains or objectively assessed cognitive impairment;

We thank the reviewer for bringing this important point to our attention regarding our inclusion criteria. While objective cognitive impairment is not formally required for participation, we employ a multi-step verification process to ensure chemotherapy-related cognitive concerns. All participants first complete a telephone screening where they must report new-onset subjective cognitive changes specifically emerging after chemotherapy, with strict exclusion of those with pre-existing complaints or neurological/psychiatric conditions. We further limit enrollment to patients not older than 65 years to reduce age-related confounders. During the baseline visit (V0), a comprehensive neuropsychological battery objectively quantifies cognitive performance, which could be used later on as a covariate. Recent evidence confirms that subjective cognitive concerns) often precedes or accompanies measurable deficits[21], supporting our approach. To better reflect this design, we have adopted the term “Post-Chemotherapy Subjective Cognitive Impairment (PCSCI) throughout the manuscript, aligning

with emerging terminology in the field while emphasizing the chemotherapy-specific context. This adjustment more accurately represents our study population and maintains clinical relevance for early intervention.

2) There is no description of how is “post-chemotherapy” assessed: will participants give information on the existence of cognitive complains before chemotherapy and breast cancer diagnosis? With this regard, please consider adding in the introduction, that many studies have presented the occurrence of cognitive impairment (in women with breast cancer and other cancers) before cancer treatment and after treatments others than chemotherapy.

We agree with the reviewer that the description of post-chemotherapy has not been complete in the previous version of the manuscript.
In short, participants will be required to provide full medical records of their chemotherapy, including any previous illnesses or diagnoses, and will be asked about cognitive complaints both before and during chemotherapy. Any diagnosed or subjective cognitive decline before chemotherapy is an exclusion criterion.

We also added information on the occurrence of cognitive complaints before and after chemotherapy. It now reads on page 4:

Cognitive impairment in cancer patients can arise from the underlying cancer itself or as a consequence of various treatments. Evidence shows that more than 20% of patients have cognitive problems even before the start of chemotherapy, which suggests the possibility of the impact of systemic inflammatory responses caused by tumor growth. Also, psychological factors such as stress, anxiety, and depression that occur after receiving a cancer diagnosis play a significant role in these early cognitive changes[22 23]. Cognitive impairment has also been reported in a wide range of cancer types and can occur as a result of various treatment methods such as surgery, radiation therapy, hormone therapy, targeted therapies, and immunotherapy[24]. In addition, factors such as increasing age, genetic background, sociodemographic conditions, and concomitant medication for other diseases can also increase the severity of this disorder and emphasize its complexity[25].

Eligibility criteria lead to a very heterogeneous study population: how long after breast cancer chemotherapy? Is the intervention designed to be administered to any women who had/has breast cancer and was treated with chemotherapy in the past, even 20 years ago? Will women who had previously another cancer or who develop another cancer after chemotherapy for breast cancer be included?

We appreciate the reviewer’s thoughtful questions regarding our eligibility criteria and sample heterogeneity. We would like to clarify and address these important points:

- Time since chemotherapy treatment:
    - Our inclusion criteria are that the chemotherapy must have been completed at least 6 months prior to participation in the clinical trial. Consequently, we will have a wide range of chemotherapy completion, so far ranging from 0.5 to 12 years, (Mean ± SD = 3.5 ± 3.3 years)
    - This window captures both subacute (6-24 months) and longer-term (2-12 years) cognitive effects while excluding acute treatment-related confounders

- Cancer history restrictions:
    - We only include female patients with breast cancer who completed their chemotherapy treatment at least 6 months ago.

- We do not include participants who had another form of cancer in the past, are currently undergoing chemotherapy, or have been diagnosed with a different form of cancer

- Cancer treatment:
  - To account for heterogeneity, we systematically document variables of interest. For example, we access the type of cancer treatment that was applied along with the chemotherapy:

Treatment	absolute frequency [n]	relative frequency [%]
Chemotherapy	26	100
Operation	26	100
Radio therapy	20	76,9
Hormone therapy	20	76.9
Antibody therapy	5	19.2

- Rationale for heterogeneity: While this introduces heterogeneity, it serves our primary study objectives to provide information
  - on the feasibility of the intervention by assessing drop-out and adherence rates and usability, and self-reported satisfaction questionnaires
  - on the effective modulation of endpoints (proof-of-concept) through the intervention (trained, non-trained function, quality of life, etc.), thus assessing a range of point estimates on the efficacy of the intervention on primary and secondary endpoints
  - guide future RCT in terms of feasibility and acceptability (adjusting the interventional approach in terms of factors increasing feasibility, e.g. by reducing drop-out and increasing adherence rates, and/or increasing compliance), safety (adjusting training and stimulation parameters to reduce occurrence of potential side effects), and maximum gain with regard to behavioral endpoints (potentially modifying certain aspects of the training tasks or defining outcomes that are sensitive to the intervention).

We added this information to the manuscript. It now reads on page 8:

Participants include right-handed women (18-65 years) with chemotherapy-treated breast cancer (completed ≥6 month prior), excluding those with other cancers, current malignancies, or pre-treatment cognitive complaints. This window captures both subacute and chronic post-chemotherapy effects while controlling for major confounders through strict neurological/psychiatric exclusions.

With such a heterogeneous population, the small sample size of 52 participants will probably be not enough to warranty two groups of participants with similar characteristics, namely those considered relevant for cognitive outcomes “education, baseline cognitive ability, neuropsychological status as well as socio-demographic and clinical characteristics” (pages 8- 9**). The authors should discuss this issue/include this limitation in the bullet points, or increase sample size.**

We agree with the reviewer that the sample size of 52 participants might reduce between-group comparability on key covariates such as education, baseline cognition, or other clinical characteristics. Please note that we account for a 13-% drop-out during the intervention phase and therefore will recruit a total number of 60 participants to ensure a sufficient number of participants to test for the tDCS effect. We now included the limitation in the bullet points under the section “Strengths and Limitations” as also suggested by the editor. In now reads:

- Randomized, double-blind, placebo-controlled design minimizes bias in intervention assessment
- Structured cognitive training protocol ensures consistency in the intervention
- Inclusion of a follow-up visit allows evaluation of long-term effects
- Monocentric trial design may increase risk of bias
- Limited sample size may reduce between-group comparability on key covariates (education, baseline cognition, clinical characteristics)

Additionally, they should explain why they decided to stratify the selection of participants based on a specific cut-off of 87% in the baseline n-back task.

We selected the 87-% accuracy cut-off for stratification based on our prior studies using identical n-back paradigms in healthy older adults [26]. This threshold was empirically determined from these previous cohorts, where it optimally differentiated: 1) ceiling effects in healthy controls (typically scoring >90%), 2) the performance range of interest in impaired populations (usually 70-85%), while 3) maintaining sufficient sensitivity to detect intervention effects. The 87% threshold specifically balances sensitivity to detect improvement in higher-functioning participants while excluding those who already perform at ceiling levels.

It now reads on page 11:
Allocation of the participants to the experimental groups will be performed by a researcher not involved in the study. Participants will be randomly allocated in a 1:1 ratio to the two groups (anodal vs. sham tDCS). Baseline performance in the n-back task will be used as strata (two performance strata; ≤ 87 % correct and > 87 % correct in the n-back task). This cut-off value was selected based on prior studies using identical n-back paradigms to balances high and low performing individuals across groups to maintain detectable of stimulation and training effects across group level.

Please clarify “double blinded”: will participants and all members of the research team, except who administer the intervention, be blinded?

We thank the reviewer for raising this important methodological question. Our study implements a carefully designed double-blind protocol in which participants and outcome assessors remain blinded to treatment allocation throughout the trial.

For participant blinding, we employ an active sham control condition that mimics the sensory experience of real tDCS. The sham protocol delivers a 30-second, 2mA current (ramping up and down) at the beginning of the session, replicating the characteristic scalp-tingling sensation of active stimulation while avoiding neuromodulatory effects. This approach ensures that participants cannot distinguish between active and sham conditions.

Regarding assessor blinding, we maintain strict separation of roles: An independent researcher who is not involved in the intervention handles the randomization and programs the NIC software (https://www.neuroelectrics.com/) with coded protocols (“A” and “B”). The

investigators involved in the intervention interact only with these blinded protocol codes and have no access to the real allocation records.

We hope to make this information clearer in the manuscript. It now reads on page 12:

As this is a double-blind trial, participants and investigators will be blinded regarding the stimulation condition. In the sham group the current will be applied for 30s to blind the participants to the intervention. In previous studies, sham tDCS has been shown as a safe and valid method of blinding participants [27-30]. At the end of the nine-session intervention, participants will be asked to state if they believe they received anodal or sham stimulation. As for the investigator blinding, a staff member who is not involved in this project will perform the randomization according to the procedure described above and provide unidentifiable labels for the two stimulation protocols (anodal, sham) for the assessor. The assessor blinding will be ensured by using NIC software (https://www.neuroelectrics.com/), where the assessor will decide between “Protocol A” and “Protocol B” that corresponds to the participants ID number and will be able to apply the stimulation accordingly without knowledge of the respective stimulation condition.

“Double-blinded” should be in the title.

We thank the reviewer for the suggestion and changed the title based on comments from reviewer 1 and reviewer 2. It now reads:

Neuromodulation through Brain Stimulation-assisted Cognitive Training in Patients with Post-Chemotherapy Subjective Cognitive Impairment (Neuromod-PCSCI) after Breast Cancer – Study Protocol of a Double-Blinded Randomized Controlled Trial

Define “excessive consumption of alcohol or nicotine on the days of intervention”.

We appreciate the reviewer’s request for clarification regarding substance use restrictions. Participants are instructed to abstain from all alcohol consumption on intervention days and to avoid excessive intake the day before the intervention (e.g., >0.1 l of wine for women). Nicotine users are permitted to maintain their typical consumption patterns to prevent withdrawal-related artifacts but are monitored for deviations. These criteria are reinforced through pre-intervention questionnaires, and individuals with a history of alcohol/drug abuse are excluded during screening.

It now reads on page 8:

Participants will be instructed to avoid excessive consumption of alcohol (e.g. < 0.1 l of wine) the day before the intervention and to abstain from all alcohol consumption on the day of the intervention. Nicotine users are permitted to maintain their typical consumption patterns to prevent withdrawal-related artifacts but are monitored for deviations. These criteria are reinforced through pre-intervention questionnaires, and individuals with a history of alcohol/drug abuse are excluded during screening. Moreover, participants. will be asked to adhere to their regular sleep schedule and to avoid drinking caffeine 90 min prior to the training sessions.

Page 9, briefly present the n-back task and give references when first referred.

We thank the reviewer for the suggestion and updated the n-back task information at their first mention. It now reads on page 8:

Secondary outcome will be working memory performance in the untrained task (nback, [31]) at follow-up assessment, operationalized by the percentage of correct responses. The n-back task (1- and 2-back) will assess working memory by requiring participants to indicate via keypress whether the current digit matches the one presented ‘n’ steps earlier. This untrained task evaluates updating, discrimination, and decision-making processes (detailed methods in Neuropsychological Assessment).

References

1. Floyd R, Dyer AH, Kennelly SP. Non-pharmacological interventions for cognitive impairment in women with breast cancer post-chemotherapy: a systematic review. *Journal of Geriatric Oncology* 2021;12(2):173-81.
2. Rapp SR, Dressler EV, Brown WM, et al. Phase III randomized, placebo-controlled clinical trial of donepezil for treatment of cognitive impairment in breast cancer survivors after adjuvant chemotherapy (WF-97116). *Journal of Clinical Oncology* 2024;42(21):2546-57.
3. Mar Fan HG, Clemons M, Xu W, et al. A randomised, placebo-controlled, double-blind trial of the effects of d-methylphenidate on fatigue and cognitive dysfunction in women undergoing adjuvant chemotherapy for breast cancer. *Supportive Care in Cancer* 2008;16:577-83.
4. Park J-H, Jung SJ, Lee LJ, et al. Impact of nonpharmacological interventions on cognitive impairment in women with breast cancer: a systematic review and meta-analysis. *Asia-Pacific Journal of Oncology Nursing* 2023;10(4):100212.
5. Yang P, Hu Q, Zhang L, et al. Effects of non-pharmacological interventions on cancer-related cognitive impairment in patients with breast cancer: A systematic review and network meta-analysis. *European Journal of Oncology Nursing* 2025:102804.
6. Campbell KL, Zadravec K, Bland KA, et al. The effect of exercise on cancer-related cognitive impairment and applications for physical therapy: systematic review of randomized controlled trials. *Physical therapy* 2020;100(3):523-42.
7. Cifu G, Power MC, Shomstein S, et al. Mindfulness-based interventions and cognitive function among breast cancer survivors: a systematic review. *BMC cancer* 2018;18:1-13.
8. Yan X, Wei S, Liu Q. Effect of cognitive training on patients with breast cancer reporting cognitive changes: a systematic review and meta-analysis. *BMJ open* 2023;13(1):e058088.
9. Garland SN, Savard J, Dalton K, et al. Rationale and protocol for a randomized waitlist controlled trial of videoconference delivered cognitive behaviour therapy for insomnia (CBT-I) to improve perceived cognitive impairment (PCI) among cancer survivors. *Contemporary Clinical Trials* 2021;103:106322.
10. Derry HM, Jaremka LM, Bennett JM, et al. Yoga and self-reported cognitive problems in breast cancer survivors: a randomized controlled trial. *Psycho‐Oncology* 2015;24(8):958-66.
11. Wei X, Yuan R, Yang J, et al. Effects of Baduanjin exercise on cognitive function and cancer-related symptoms in women with breast cancer receiving chemotherapy: a randomized controlled trial. *Supportive Care in Cancer* 2022;30(7):6079-91.
12. Tong T, Pei C, Chen J, et al. Efficacy of acupuncture therapy for chemotherapy-related cognitive impairment in breast cancer patients. *Medical science monitor: international medical journal of experimental and clinical research* 2018;24:2919.
13. Vance DE, Frank JS, Bail J, et al. Interventions for cognitive deficits in breast cancer survivors treated with chemotherapy. *Cancer nursing* 2017;40(1):E11-E27.
14. Oldacres L, Hegarty J, O'Regan P, et al. Interventions promoting cognitive function in patients experiencing cancer related cognitive impairment: A systematic review. *Psycho‐Oncology* 2023;32(2):214-28.

15. Woods AJ, Antal A, Bikson M, et al. A technical guide to tDCS, and related non-invasive brain stimulation tools. *Clin Neurophysiol* 2016;127(2):1031-48. doi: 10.1016/j.clinph.2015.11.012 [published Online First: 20151122]
16. Jacobson L, Koslowsky M, Lavidor M. tDCS polarity effects in motor and cognitive domains: a meta-analytical review. *Exp Brain Res* 2012;216(1):1-10. doi: 10.1007/s00221-011-2891-9 [published Online First: 20111012]
17. Polania R, Nitsche MA, Ruff CC. Studying and modifying brain function with non-invasive brain stimulation. *Nat Neurosci* 2018;21(2):174-87. doi: 10.1038/s41593-017-0054-4 [published Online First: 20180108]
18. Vergallito A, Feroldi S, Pisoni A, et al. Inter-Individual Variability in tDCS Effects: A Narrative Review on the Contribution of Stable, Variable, and Contextual Factors. *Brain Sci* 2022;12(5) doi: 10.3390/brainsci12050522 [published Online First: 20220420]
19. Knecht S, Drager B, Deppe M, et al. Handedness and hemispheric language dominance in healthy humans. *Brain* 2000;123 Pt 12:2512-8. doi: 10.1093/brain/123.12.2512
20. Webster K, Cella D, Yost K. The Functional Assessment of Chronic Illness Therapy (FACIT) Measurement System: properties, applications, and interpretation. *Health Qual Life Outcomes* 2003;1:79. doi: 10.1186/1477-7525-1-79 [published Online First: 20031216]
21. Jessen F, Amariglio RE, Buckley RF, et al. The characterisation of subjective cognitive decline. *Lancet Neurol* 2020;19(3):271-78. doi: 10.1016/S1474-4422(19)30368-0 [published Online First: 20200117]
22. Dijkshoorn AB, van Stralen HE, Sloots M, et al. Prevalence of cognitive impairment and change in patients with breast cancer: a systematic review of longitudinal studies. *Psycho‐Oncology* 2021;30(5):635-48.
23. van der Willik KD, Hauptmann M, Jóźwiak K, et al. Trajectories of cognitive function prior to cancer diagnosis: a population-based study. *JNCI: Journal of the National Cancer Institute* 2020;112(5):480-88.
24. Joly F, Giffard B, Rigal O, et al. Impact of cancer and its treatments on cognitive function: advances in research from the Paris International Cognition and Cancer Task Force Symposium and update since 2012. *Journal of pain and symptom management* 2015;50(6):830-41.
25. Ahles TA, Root JC. Cognitive effects of cancer and cancer treatments. *Annual review of clinical psychology* 2018;14(1):425-51.
26. Antonenko D, Thams F, Grittner U, et al. Randomized trial of cognitive training and brain stimulation in non-demented older adults. *Alzheimers Dement (N Y)* 2022;8(1):e12262. doi: 10.1002/trc2.12262 [published Online First: 20220223]
27. Floel A, Cohen LG. Recovery of function in humans: Cortical stimulation and pharmacological treatments after stroke. *Neurobiology of Disease* 2010;37(2):243-51. doi: 10.1016/j.nbd.2009.05.027
28. Gandiga PC, Hummel FC, Cohen LG. Transcranial DC stimulation (tDCS): A tool for double-blind sham-controlled clinical studies in brain stimulation. *Clinical Neurophysiology* 2006;117(4):845-50. doi: https://doi.org/10.1016/j.clinph.2005.12.003
29. Kuo HI, Bikson M, Datta A, et al. Comparing cortical plasticity induced by conventional and high-definition 4 × 1 ring tDCS: a neurophysiological study. *Brain stimulation* 2013;6(4):644-8. doi: 10.1016/j.brs.2012.09.010 [published Online First: 2012/11/15]
30. Schlaug G, Renga V. Transcranial direct current stimulation: a noninvasive tool to facilitate stroke recovery. *Expert review of medical devices* 2008;5(6):759-68.
31. Lezak MD, Howieson DB, Bigler ED, et al. Neuropsychological Assessment. 5th edition ed. Oxford ; New York: Oxford University Press 2012.
